# Fibroblasts repair blood-brain barrier damage and hemorrhagic brain injury via TIMP2

**DOI:** 10.1016/j.celrep.2022.111709

**Published:** 2022-11-22

**Authors:** Lingling Xu, Abhijit Nirwane, Ting Xu, Minkyung Kang, Karan Devasani, Yao Yao

**Affiliations:** 1 Department of Molecular Pharmacology and Physiology, Morsani College of Medicine, University of South Florida, 12901 Bruce B. Downs Blvd., MDC 8, Tampa, FL 33612, USA; 2 Department of Pharmaceutical and Biomedical Sciences, University of Georgia, Athens, GA 30602, USA; 3 Lead contact

## Abstract

The function of fibroblasts in intracerebral hemorrhage (ICH) remains elusive. By targeting Col1α1, a fibroblast-specific marker, we generate mice with ablated Col1α1^+^ fibroblasts. These mutants show exacerbated blood-brain barrier (BBB) damage, enlarged injury volume, and worse neurological function, highlighting a beneficial role of Col1α1^+^ fibroblasts in ICH. Echoing these findings, fibroblasts significantly decrease endothelial permeability in an *in vitro* ICH model. Next, we demonstrate that fibroblasts promote BBB integrity in ICH mainly via up-regulating tight junction proteins without affecting transcytosis-associated proteins, indicating a paracellular rather than transcellular mechanism. A subsequent mechanistic study reveals that the BBB-protective effect of fibroblasts is partially mediated by TIMP metallopeptidase inhibitor 2 (TIMP2). Furthermore, we find that exogenous TIMP2 attenuates BBB disruption in these mutants after ICH. These results suggest that Col1α1^+^ fibroblasts repair BBB damage in ICH via the paracellular pathway in a TIMP2-dependent manner, and that Col1α1^+^ fibroblasts and TIMP2 may be targeted in ICH treatment.

## In brief

Xu et al. investigate the functional significance of Col1α1^+^ fibroblasts in intracerebral hemorrhage. They show that ablation of Col1α1^+^ fibroblasts exacerbates hemorrhagic brain injury and blood-brain barrier damage through the paracellular mechanism. They further demonstrate that Col1α1^+^ fibroblasts exert this beneficial function partially via TIMP2.

## Graphical Abstract

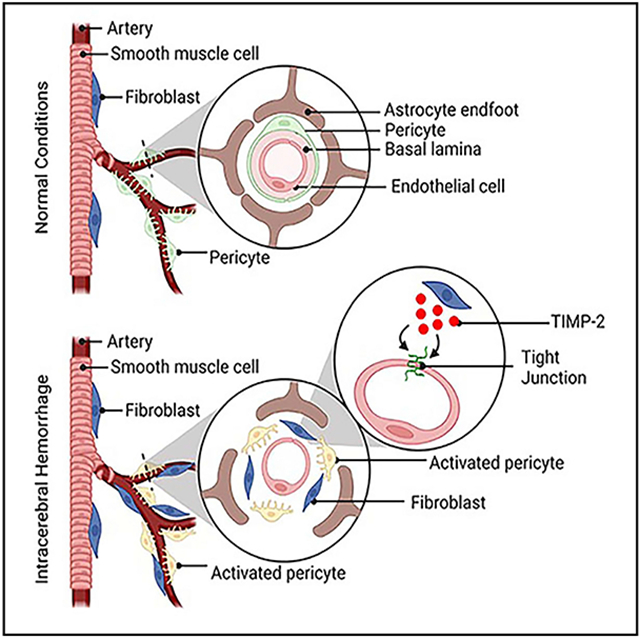

## INTRODUCTION

Fibroblasts, a heterogeneous cell population, are involved in many important functions, such as wound healing.^1-3^ In the CNS, fibroblasts are mainly located in the meninges and perivascular space.^4^ Mounting evidence suggests that fibroblasts become activated and contribute to the formation of fibrotic scar in various neurological disorders, including experimental autoimmune encephalomyelitis (EAE), spinal cord injury (SCI), ischemic stroke, and sporadic amyotrophic lateral sclerosis.^4-9^ The functional significance of fibroblast activation and the roles of fibrotic scar, however, are controversial. On one hand, it has been reported that fibroblasts exert a detrimental function. For example, fibroblast-derived extracellular matrix (ECM) proteins in scar tissue inhibit neuronal regeneration after CNS injury.^10-12^ Consistent with these findings, ablation of proliferating Col1α2-expressing fibrotic cells leads to increased oligodendrocyte lineage cells and improved motor function in the chronic stage of EAE.^7^ On the other hand, there is also evidence suggesting a beneficial role of fibroblasts. For instance, platelet-derived growth factor receptor alpha (PDGFRα)^+^ fibroblasts protect blood-brain barrier (BBB) integrity and reduce hemorrhagic transformation at the subacute stage of ischemic stroke.^13^ In addition, inhibition of scar formation by blocking the generation of “type A” pericytes, which possibly contain fibroblasts, results in failure of tissue sealing in an SCI model.^14^

These controversial findings may be explained by the lack of fibroblast-specific markers. Although several markers, including PDGFRα, PDGFRβ, and Col1α2, have been used to identify fibroblasts,^7,8,13^ none of these markers are fibroblast specific. For example, PDGFRα labels both fibroblasts and oligodendrocyte precursor cells.^15^ PDGFRβ and Col1α2 mark both fibroblasts and mural cells.^4,16^ It is likely that the “fibroblast” populations in previous studies contain contaminating cells, such as mural cells, which are also highly responsive to CNS injury. Recent single-cell RNA sequencing (RNA-seq) analysis revealed that Col1α1 is selectively expressed in fibroblasts, but not other cells in the brain,^16^ indicating that Col1α1 may be used to label fibroblasts specifically.

Intracerebral hemorrhage (ICH) accounts for 10%–30% of all strokes and is the subtype with the highest rate of mortality and morbidity.^17-19^ Unfortunately, there is no effective treatment for ICH partially due to an incomplete understanding of ICH pathogenesis. Although fibroblasts become activated and promote BBB integrity in ischemic stroke,^13,20^ their function in ICH remains unclear.

In this study, we reported a beneficial role of Col1α1^+^ fibroblasts in ICH. We further showed that Col1α1^+^ fibroblasts promoted BBB repair after ICH through the paracellular pathway in a TIMP metallopeptidase inhibitor 2 (TIMP2)-dependent manner. These findings suggest that Col1α1^+^ fibroblasts and TIMP2 may be targeted in the treatment of ICH.

## RESULTS

### Col1α1^+^ fibroblasts are not ablated in FKO mice under homeostatic conditions

**Figure 1.**
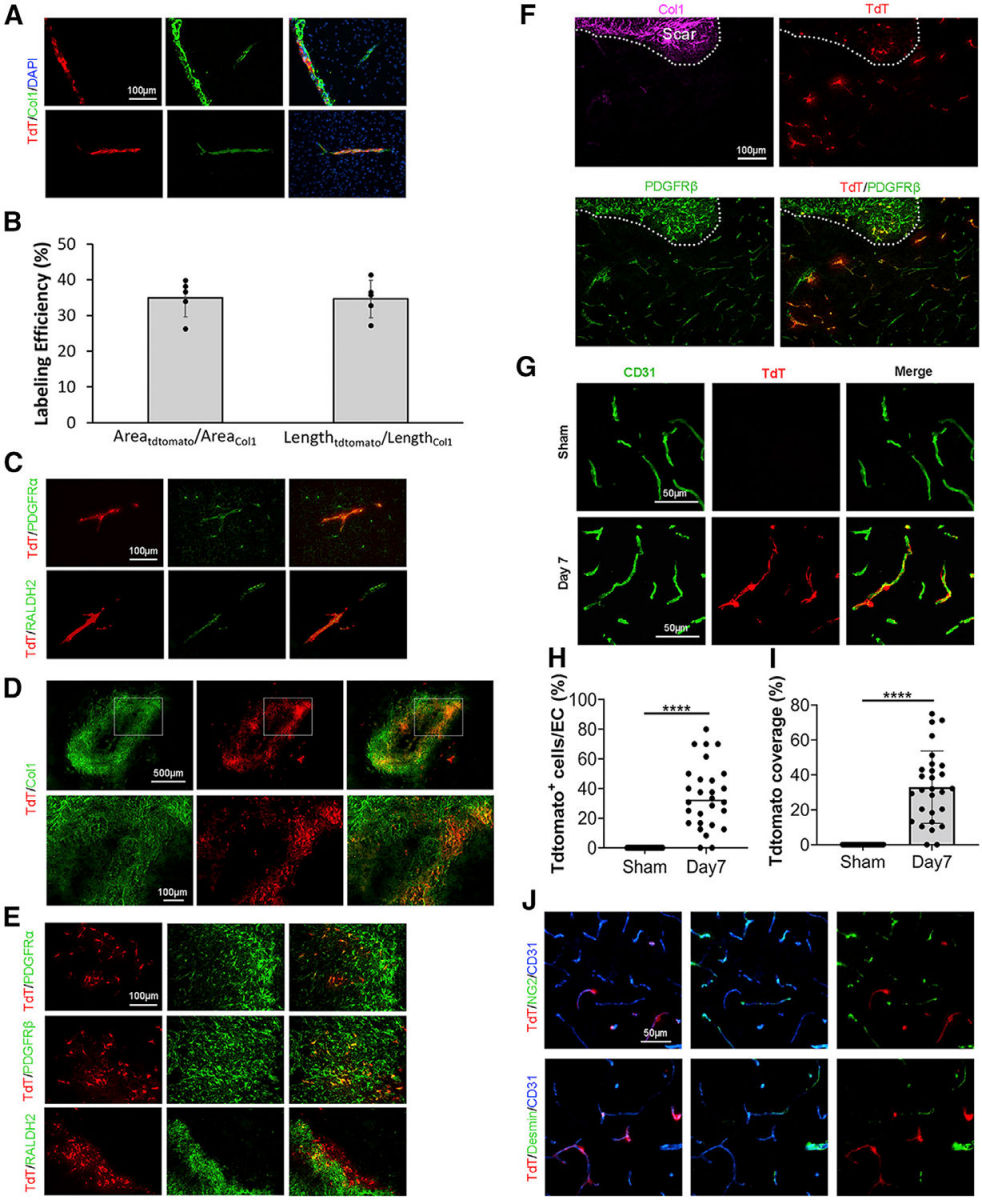
Lineage tracing of Col1α1^+^ fibroblasts (A) Representative images of tdTomato (TdT; red), Col1 (green), and DAPI (blue) in Col1α1-tdTomato brains under homeostatic conditions. Scale bar, 100 μm. (B) Quantification of labeling efficiency using both vessel area and vessel length in Col1α1-tdTomato brains under homeostatic conditions. n = 5 mice. (C) Representative images of TdT (red), PDGFRα (green), and RALDH2 (green) in Col1α1-tdTomato brains under homeostatic conditions. Scale bar, 100 μm. (D) Representative images of TdT (red) and Col1 (green) in Col1α1-tdTomato brains at day 7 after ICH. Scale bars, 500 μm (upper panel); 100 μm (lower panel). (E) Representative images of TdT (red), PDGFRα (green), PDGFRβ (green), and RALDH2 (green) in the peri-hematoma region of Col1α1-tdTomato mice at day 7 after ICH. Scale bar, 100 μm. (F) Representative images of TdT (red), PDGFRβ (green), and Col1 (magenta) in the peri-hematoma region in Col1α1-tdTomato mice at day 7 after ICH. Scale bar, 100 μm. (G) Representative images of TdT (red) and CD31 (green) in the peri-hematoma region in Col1α1-tdTomato mice in sham group and at day 7 after ICH. Scale bars, 50 μm. (H) Quantification of the ratio of TdT-positive cells to endothelial cells in Col1α1-tdTomato brains in the sham group and at day 7 after ICH. n = 28 sections from 3 mice. ****p < 0.0001 by Student’s t test. (I) Quantification of tdTomato coverage over CD31^+^ capillaries in Col1α1-tdTomato mice in sham group and at day 7 after ICH. n = 28 sections from 3 mice. ****p < 0.0001 by Student’s t test. (J) Representative images of TdT (red), NG2 (green), Desmin (green), and CD31 (blue) in the peri-hematoma region in Col1α1-tdTomato mice at day 7 after ICH. Scale bar, 50 μm. Data were shown as mean ± SD. ICH, intracerebral hemorrhage. See also Figures S1-S4.

A recent single-cell RNA-seq analysis showed that Col1α1 is expressed specifically by fibroblasts in the CNS.^16^ To target these fibroblasts selectively, we used Col1α1-Cre mice in this study. First, we performed a lineage-tracing study using Ai14^+/−^:Col1α1-Cre^+^ (Col1α1-tdTomato) mice. Consistent with the single-cell RNA-seq data, tdTomato^+^ cells were mainly found in the meninges and large blood vessels under homeostatic conditions (Figure 1A). It should be noted, however, that not all Col1^+^ blood vessels expressed tdTomato (Figure 1A). Quantifications showed that 34.9% of Col1^+^ vessel area and 34.7% of Col1^+^ vessel length expressed tdTomato (Figure 1B), suggesting that the *Col1α1* promoter may be weak under normal conditions. In addition, tdTomato also merged with PDGFRα in blood vessels, but not brain parenchyma (Figure 1C), and partially colocalized with fibroblast marker RALDH2 (Figure 1C). Together, these resuits suggest that Col1α1-Cre specifically labels a subpopulation of fibroblasts.

To investigate the functions of Col1α1^+^ fibroblasts, we performed loss-of-function studies using mice expressing diphtheria toxin receptor (DTR) in Col1α1^+^ fibroblasts (DTR^+/−^:Col1α1-Cre^+^, termed FKO). To ablate these Col1α1^+^ fibroblasts, we intraperitoneally injected FKO mice and controls with diphtheria toxin (DT, 500 ng) daily for 5 consecutive days and analyzed 24 h after the last injection (Figure S1A). Both FKO mice with saline injection and DTR^+/−^ littermates with DT injection were used as controls. Interestingly, FKO mice and the controls showed comparable Col1 expression in the brain under homeostatic conditions (Figures S1B and S1C). As a stable ECM protein, Col1 may not be able to accurately reflect fibroblast number. Thus, we also examined fibroblast number by quantifying PDGFRα^+^Olig2^−^ cells and found no difference in control and FKO brains (Figures S1D and S1E). In addition, we further conducted *in situ* hybridization analysis and observed similar numbers of *Col1α1* (mRNA)-positive cells between control and FKO mice (Figures S1F and S1G). These findings suggest that Col1α1^+^ fibroblasts are not efficiently ablated in FKO mice under homeostatic conditions.

One possible explanation is that intraperitoneally injected DT is unable to cross the BBB to ablate fibroblasts in the CNS. To explore this possibility, we directly administered DT into the lateral ventricle of FKO mice using osmotic pumps. Interestingly, control and FKO mice exhibited comparable Col1 levels (Figures S2A and S2B), PDGFRα^+^Olig2^−^ cells (Figures S2C and S2D), and *Col1α1*^+^ cells (Figures S2E and S2F). These results strongly suggest that the failure of fibroblast ablation under homeostatic conditions is not due to lack of CNS infiltration of intraperitoneally injected DT. Instead, it is probably due to weak activity of the *Col1α1* promoter under homeostatic conditions.

Consistent with the lack of fibroblast ablation, FKO mice were grossly normal under homeostatic conditions. Functional studies revealed negligible levels of biotin (Figure S3A) and hemoglobin (Figure S3B) in FKO brains under homeostatic conditions, indicating intact BBB integrity. Echoed with these results, comparable levels of tight junction proteins (TJPs), including zona occludens 1 (ZO-1; Figures S3C and S3D) and occludin (Figures S3E and S3F), and pericyte coverage (Figures S3G and S3H) were found in control and FKO brains. Again, these results suggest that Col1α1^+^ fibroblasts are not ablated in FKO mice under homeostatic conditions.

### Col1α1^+^ fibroblasts are ablated in FKO mice after ICH

Because fibroblasts become activated after various types of injury,^4^ the phenotype of FKO mice was further characterized in the ICH model. Similarly, we first performed a lineage-tracing study using Col1α1-tdTomato mice after ICH. Although few tdTomato^+^ cells were detected at day 2 after injury, these tdTomato^+^ cells were substantially increased at the injury site on day 5 after ICH (Figure S4A). By day 7 after ICH, tdTomato^+^ cells accumulated in the peri-hematoma area, forming a scar-like structure (Figure S4A). Consistent with this result, Col1 displayed a similar expression pattern after ICH (Figures S4B and S4C). Immunohistochemical analyses showed that tdTomato co-localized with multiple fibroblast markers, including Col1, PDGFRα, PDGFRβ, and RALDH2 in the peri-hematoma area (Figures 1D and 1E), indicating a fibroblast nature of these cells. In the region immediately outside the scar, however, tdTomato co-localized with PDGFRβ, but not Col1 (Figure 1F). Additionally, tdTomato signal was associated with small blood vessels in this peri-scar region (Figure 1F). Further analyses showed that tdTomato^+^ fibroblasts accumulated in capillaries at day 7 after ICH, but not in sham controls (Figures 1G-1I). Interestingly, these tdTomato^+^ cells did not express typical mural cell markers, including NG2 and Desmin (Figure 1J). Together, these results suggest that Col1α1^+^ fibroblasts may migrate to capillaries after ICH and are likely involved in the repair of vascular integrity.

**Figure 2.**
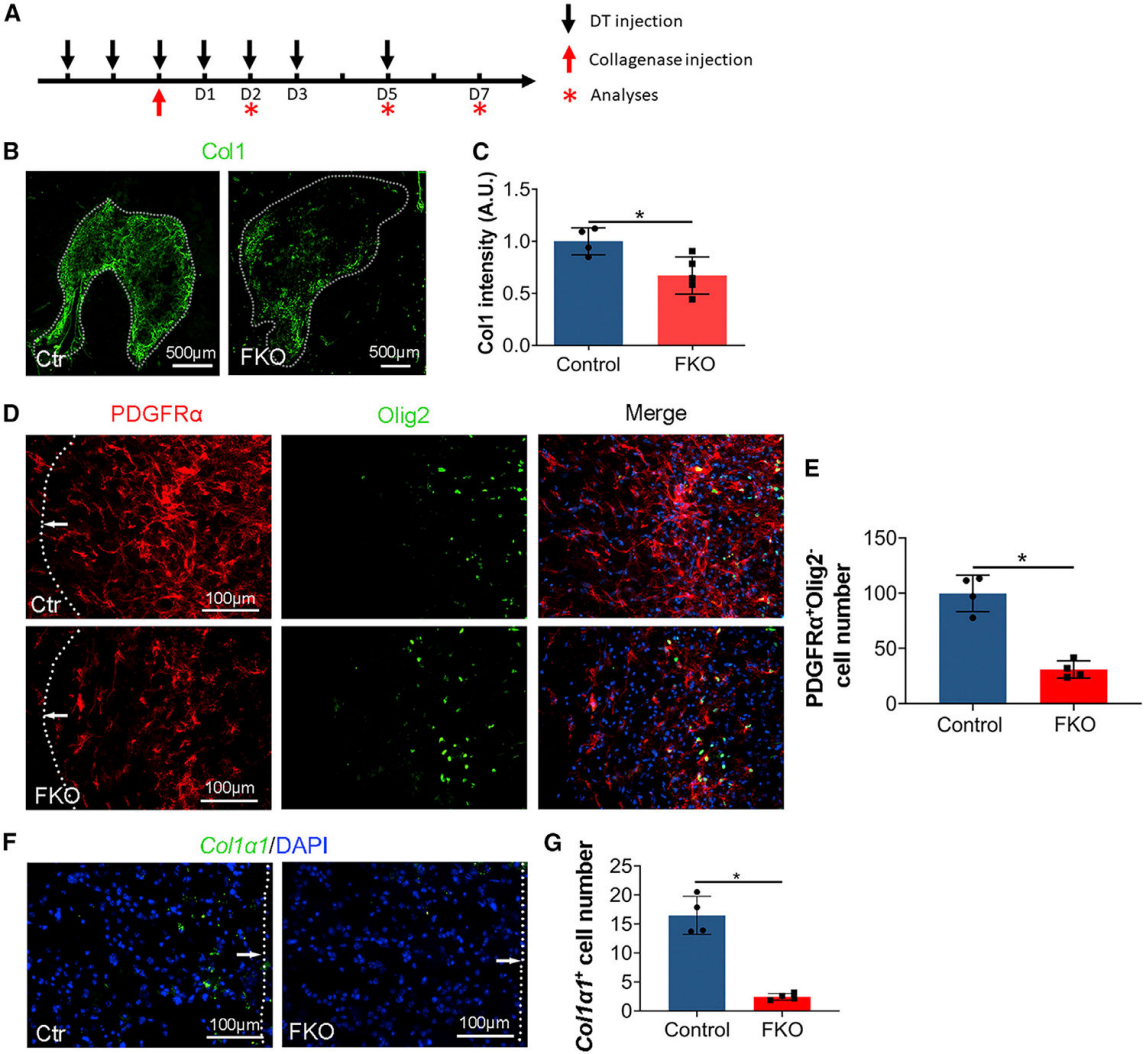
Col1α1^+^ fibroblasts are ablated in FKO mice after ICH (A) Schematic illustration of time points for DT and collagenase injections. (B) Representative images of Col1 (green) in control and FKO mice at day 7 after ICH. Dotted lines indicate hematoma. Scale bars, 500 μm. (C) Quantification of Col1 intensity in the peri-hematoma region in control and FKO mice. n = 4–5 mice. *p = 0.0317 by Mann-Whitney U test. (D) Representative images of PDGFRα (red), Olig2 (green), and DAPI (blue) in control and FKO mice at day 7 after ICH. Dotted lines and arrows indicate hematoma. Scale bars, 100 μm. (E) Quantification of PDGFRα^+^Olig2^−^ cells in the peri-hematoma region in control and FKO mice. n = 4 mice. *p = 0.0286 by Mann-Whitney U test. (F) Representative images of *Col1α1* mRNA (green, detected by RNAscope *in situ* hybridization) and DAPI (blue) in control and FKO mice at day 7 after ICH. Dotted lines and arrows indicate hematoma. Scale bars, 100 μm. (G) Quantification of *Col1α1* mRNA-expressing cells in the peri-hematoma region in control and FKO mice. n = 4 mice. *p = 0.0286 by Mann-Whitney U test. Data were shown as mean ± SD. See also Figure S4.

Next, we examined fibroblast ablation efficiency in FKO mice after ICH. DT was administered on days −2, −1, 0, 1, 2, 3, and 5 after ICH (Figure 2A). A time-course study revealed significantly reduced Col1 levels at the injury site in FKO brains at days 5 and 7, but not day 2 after ICH, compared with the controls (Figures 2B, 2C, S4B, and S4C). To more accurately quantify fibroblast number in the brain, we performed co-staining against PDGFRα and Olig2. Compared with the controls, FKO mice exhibited an ~69% decrease of PDGFRα^+^Olig2^−^ fibroblasts at the injury site at day 7 after ICH (Figures 2D and 2E). In addition, we also conducted *in situ* hybridization to detect *Col1α1* expression at the injury site and found a dramatically decreased number of *Col1α1*^+^ cells in FKO mice (Figures 2F and 2G). These findings suggest that Col1α1^+^ fibroblasts are substantially ablated in FKO mice after ICH.

### Ablation of Col1α1^+^ fibroblasts aggravates ICH outcome

**Figure 3.**
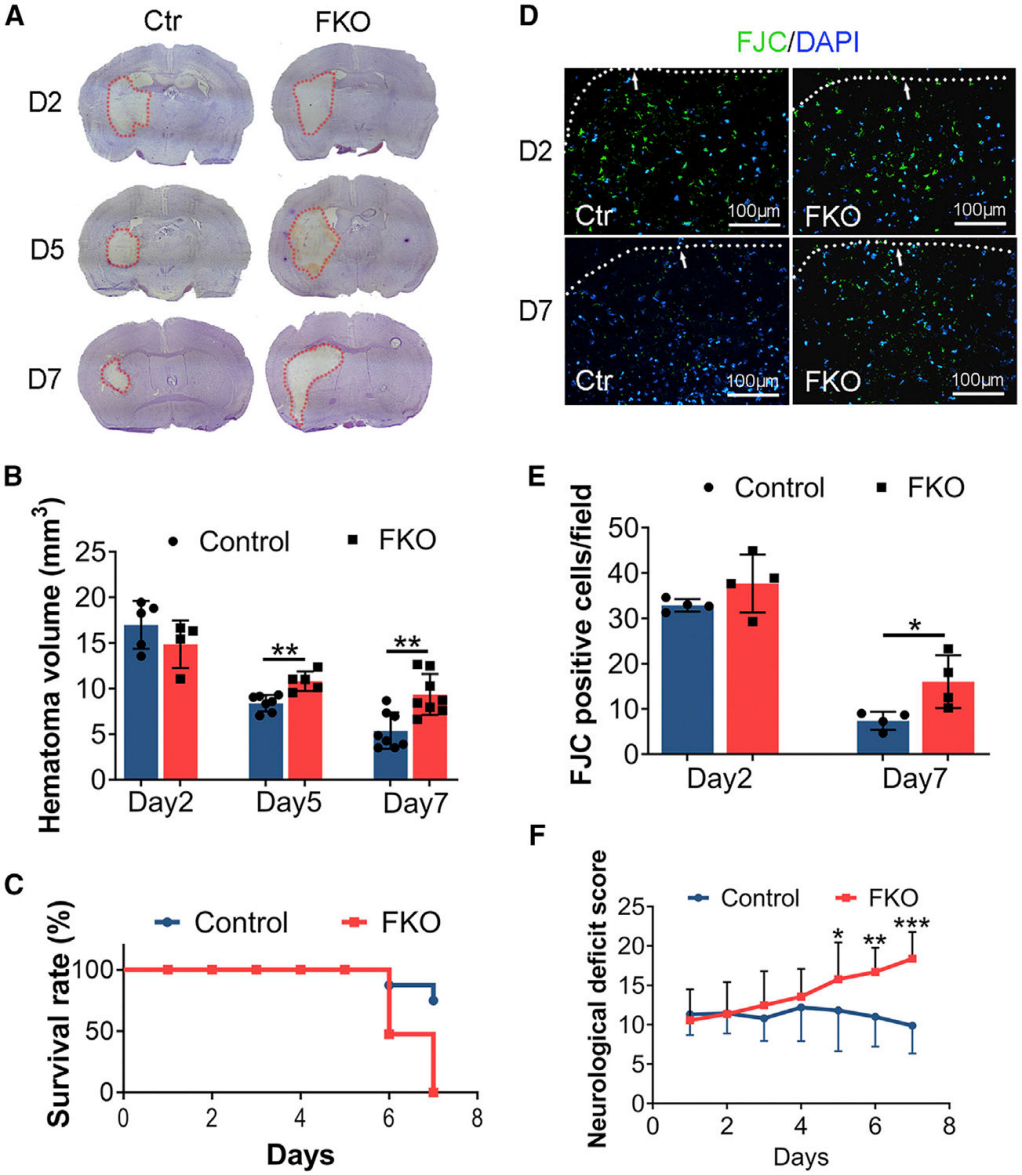
Ablation of Col1α1^+^ fibroblasts aggravates ICH outcome (A) Representative images of cresyl violet staining in control and FKO brains at days 2, 5, and 7 after ICH. Dotted lines indicate hematoma. (B) Quantification of injury volume in control and FKO mice at days 2, 5, and 7 after ICH. n = 4–5 mice for day 2;n = 5–7 mice for day 5; and n = 8 mice for day 7. **p = 0.0017 and **p = 0.0022 at days 5 and 7 after ICH by Student’s t test, respectively. (C) Survival rate for control and FKO mice after ICH. n = 16 and 19 mice for control and FKO mice, respectively. (D) Representative images of FJC (green) and DAPI (blue) in control and FKO brains at days 2 and 7 after ICH. Dotted lines and arrows indicate hematoma. Scale bars, 100 μm. (E) Quantification of FJC^+^ cells in the peri-hematoma region in control and FKO mice. n = 4 mice. *p = 0.0286 by Mann-Whitney U test. (F) Neurological deficit score in control and FKO mice after ICH. n = 16–19 mice for days 1–5 and n = 8–10 mice for days 6 and 7. *p = 0.0269, **p = 0.0027, ***p = 0.0002 by Student’s t test. Data were shown as mean ± SD. FJC, fluor jade C. See also Videos S1 and S2.

Hematoma volume actively contributes to ICH outcome.^21^ Control and FKO mice displayed similar hematoma size at day 2 after ICH (Figures 3A and 3B), indicating comparable initial injury. At days 5 and 7 after injury, however, FKO mice showed significantly larger injury volume (Figures 3A and 3B). Consistent with hematoma size, FKO mice exhibited significantly decreased survival rate (Figure 3C) and more fluor jade C-positive (FJC^+^) degenerating neurons at day 7 after injury (Figures 3D and 3E), suggesting exacerbated neuronal death. Although control mice were able to move around at day 6 after injury (Video S1), FKO mice failed to do so (Video S2). Echoed with this observation, FKO mice exhibited significantly higher neurological deficit scores in the subacute phase after ICH compared with the controls (Figure 3F), indicating worse neurological function. Together, these results highlight a beneficial role of Col1α1^+^ fibroblasts in ICH.

### Ablation of Col1α1^+^ fibroblasts exacerbates BBB damage in the subacute phase of ICH

**Figure 4.**
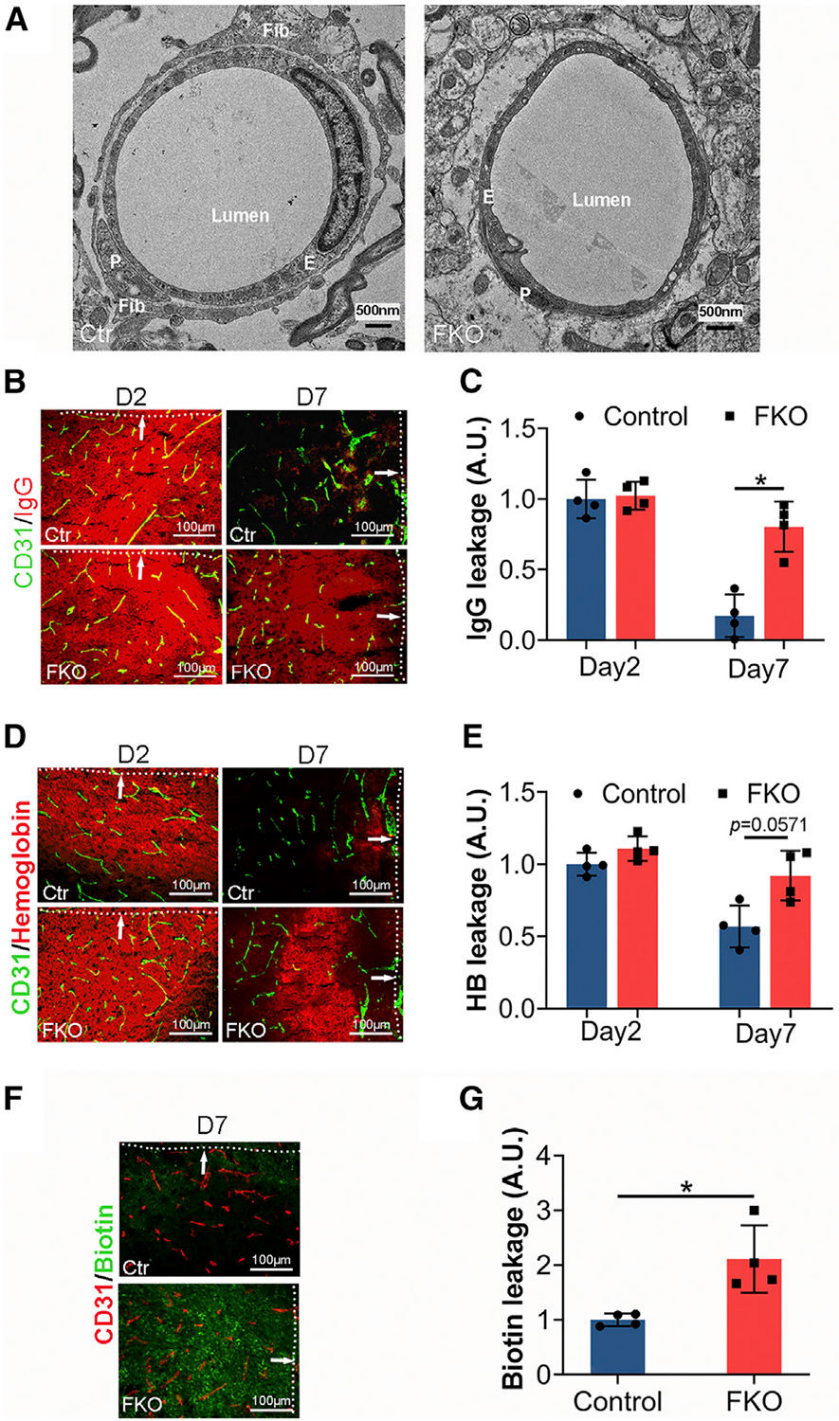
Ablation of Col1α1^+^ fibroblasts exacerbates BBB damage (A) Representative transmission electron microscopy images showing association of fibroblasts with capillaries in control brains and lack of fibroblasts around capillaries in FKO brains at day 7 after ICH. Fibroblasts (Fib), endothelial cells (E), and pericytes (P) are indicated in the images. Scale bars, 500 nm. (B) Representative images of IgG (red) and CD31 (green) in the peri-hematoma region in control and FKO brains at days 2 and 7 after ICH. Dotted lines and arrows indicate hematoma. Scale bars, 100 μm. (C) Quantification of IgG intensity in the peri-hematoma region in control and FKO brains at days 2 and 7 after ICH. n = 4 mice. *p = 0.0286 by Mann-Whitney U test. (D) Representative images of hemoglobin (red) and CD31 (green) in the peri-hematoma region in control and FKO brains at days 2 and 7 after ICH. Dotted lines and arrows indicate hematoma. Scale bars, 100 μm. (E) Quantification of hemoglobin intensity in the peri-hematoma region in control and FKO brains at days 2 and 7 after ICH. n = 4 mice. p = 0.0571 by Mann-Whitney U test. (F) Representative images of biotin (green) and CD31 (red) in the peri-hematoma region in control and FKO brains at day 7 after ICH. Dotted lines and arrows indicate hematoma. Scale bars, 100 μm. (G) Quantification of biotin intensity in the peri-hematoma region in control and FKO brains at day 7 after ICH. n = 4 mice. *p = 0.0286 by Mann-Whitney U test. Data were shown as mean ± SD. See also Figures S5 and S6.

Consistent with the immunohistochemical data, we frequently observed fibroblasts that extended their processes to wrap capillaries in control, but not FKO, brains at day 7 after ICH under transmission electronic microscopy (Figure 4A), again suggesting a possible role of Col1α1^+^ fibroblasts in BBB repair after ICH. Because BBB disruption contributes to secondary brain damage and correlates with ICH outcome,^22^ we next examined BBB integrity in FKO and control mice after ICH using endogenous markers. Similar levels of IgG (Figures 4B and 4C) and hemoglobin (Figures 4D and 4E) were found in FKO and control brains at day 2 after ICH, again indicating comparable initial injury in these mice. By day 7 after ICH, however, dramatically increased IgG (Figures 4B and 4C) and hemoglobin (Figures 4D and 4E) were observed in FKO brains, suggesting more severe BBB disruption. Consistent with these findings, FKO mice displayed enhanced leakage of exogenous tracer biotin at day 7 after ICH, compared with the controls (Figures 4F and 4G). These results suggest that Col1α1^+^ fibroblasts play an important role in BBB repair after ICH.

### Ablation of Col1α1^+^ fibroblasts increases paracellular leakage

**Figure 5.**
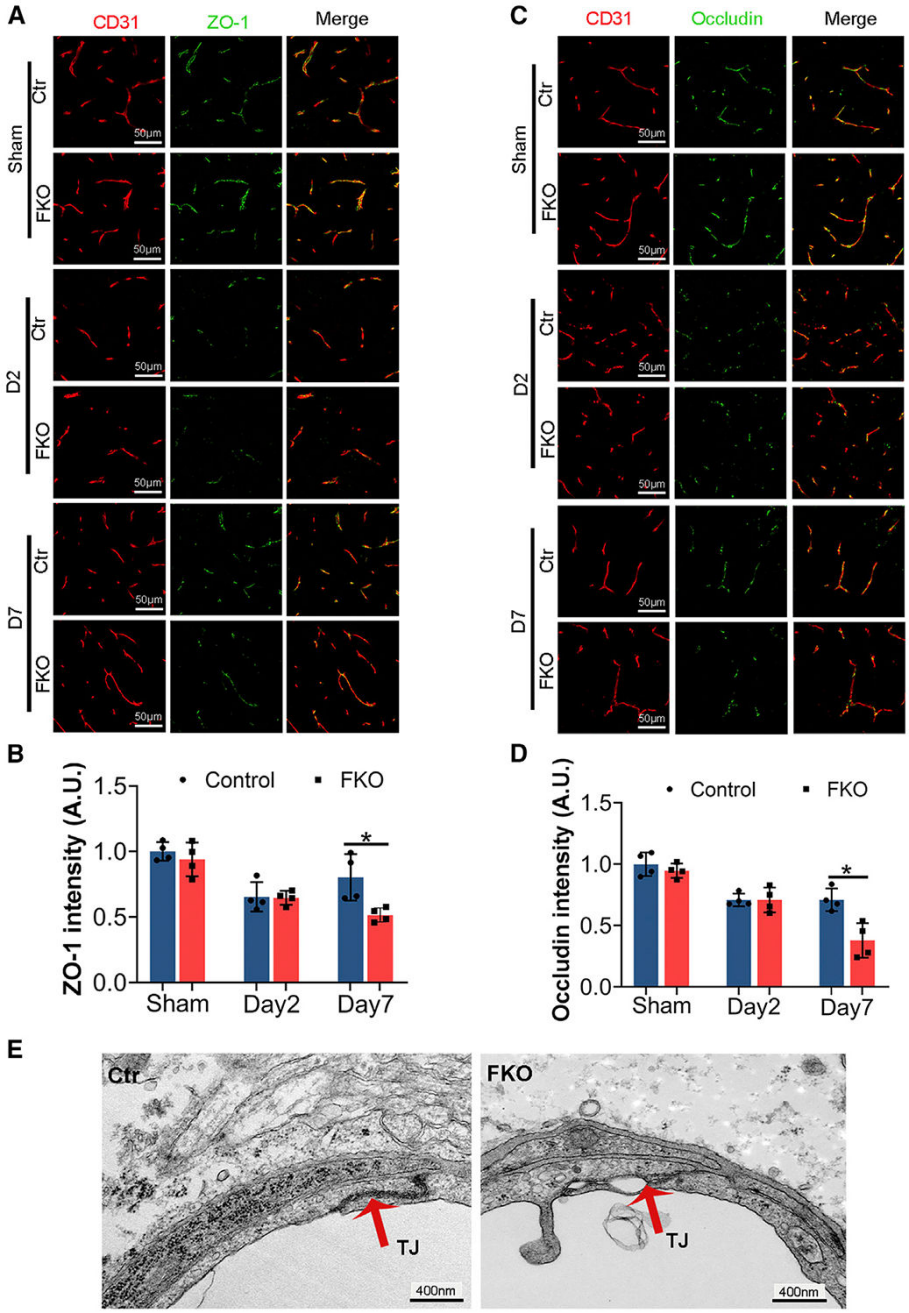
Ablation of Col1α1^+^ fibroblasts increases tight junction disruption (A) Representative images of ZO-1 (green) and CD31 (red) in the peri-hematoma region in control and FKO brains in the sham group and at days 2 and 7 after ICH. Scale bars, 50 μm. (B) Quantification of ZO-1 intensity in the peri-hematoma region in control and FKO brains. n = 4 mice, *p = 0.0286 by Mann-Whitney U test. (C) Representative images of occludin (green) and CD31 (red) in the peri-hematoma region in control and FKO brains in the sham group and at days 2 and 7 after ICH. Scale bars, 50 μm. (D) Quantification of occludin intensity in the peri-hematoma region in control and FKO brains. n = 4 mice, *p = 0.0286 by Mann-Whitney U test. (E) Representative transmission electron microscopy images showing the ultrastructure of endothelial tight junctions in control and FKO brains at day 7 after ICH. Red arrows indicate tight junctions. Scale bars, 400 nm. Data were shown as mean ± SD.

Both paracellular and transcellular mechanisms contribute to the barrier function of BBB. Paracellular integrity is formed by tight junctions, where TJPs seal gaps between endothelial cells.^23,24^ To determine whether aggravated paracellular leakage is responsible for the enhanced BBB disruption in FKO mice after ICH, we examined the expression of two TJPs (ZO-1 and occludin) by immunohistochemistry. Although FKO and control mice showed similar levels of ZO-1 and occludin in the sham group and at day 2 after ICH, both TJPs were dramatically reduced in FKO mice at day 7 after ICH (Figures 5A-5D). Consistent with these biochemical changes, ultrastructural alteration of the tight junctions was observed in FKO mice at day 7 after ICH. Specifically, tight junctions were electron dense and well formed in control brains but became electron light and disrupted in FKO brains (Figure 5E). These results suggest that Col1α1^+^ fibroblasts repair BBB integrity after ICH by regulating TJP expression and tight junction structure.

### Ablation of Col1α1^+^ fibroblasts fails to affect transcellular leakage

Transcellular integrity is maintained by low rate of transcytosis, which is predominantly mediated by meca32 and caveolin-1.^25,26^ To determine whether increased transcytosis is responsible for the exacerbated BBB leakage in FKO mice after ICH, we assessed the expression of meca32 and caveolin-1 by immunohistochemistry. Control and FKO mice showed comparable meca32 expression at day 7 after ICH (Figures S5A and S5B). In addition, similar levels of endothelial caveolin-1 were observed in control and FKO mice at day 7 after ICH (Figures S5C and S5D). Echoed with these results, transmission electron microscopy revealed comparable numbers of endothelial pinocytotic vesicles in control and FKO mice at day 7 after ICH (Figures S5E and S5F). These results suggest that the transcellular pathway plays a minimal role in Col1α1^+^ fibroblast-mediated BBB repair at the subacute phase after ICH.

### Ablation of Col1α1^+^ fibroblasts affects pericyte coverage but not astrocyte polarity

Accumulating evidence suggests that both pericyte coverage^27,28^ and astrocyte polarity^29^ actively contribute to BBB integrity. We first examined pericyte coverage in control and FKO mice after ICH. Although unaffected in the sham group and at day 2 after ICH, PDGFRβ intensity (Figures S6A and S6B) and PDGFRβ coverage on CD31^+^ capillaries (Figures S6A and S6C) dramatically decreased in FKO mice at day 7 after ICH compared with controls. Similar results were observed when CD13 and podocalyxin were used as pericyte and vascular markers, respectively (Figures S6D-S6F). These results suggest that Col1α1^+^ fibroblasts enhance pericyte coverage after ICH. Next, we examined whether fibroblast ablation affects astrocyte polarity by performing aquaporin-4 (AQP4) and CD31 co-staining. Although AQP4 expression and coverage were substantially reduced at the peri-hematoma region in both control and FKO brains at days 2 and 7 after ICH, no significant difference was observed between genotypes (Figures S6G-S6I), suggesting a minimal role of Col1α1^+^ fibroblasts in astrocyte polarity after ICH.

### Fibroblasts promote endothelial barrier integrity in an *in vitro* ICH model

**Figure 6.**
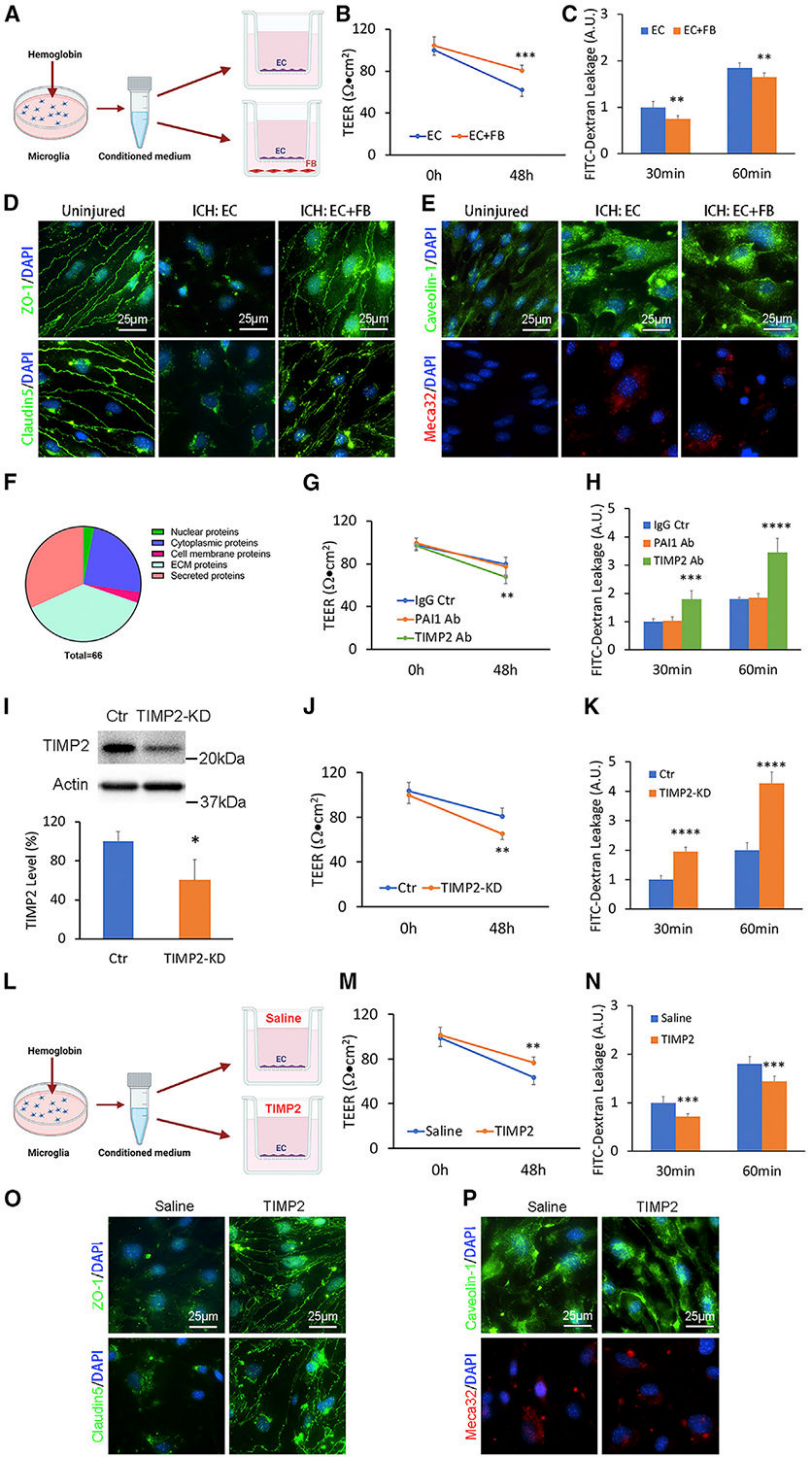
Fibroblast-derived TIMP2 promotes BBB integrity in an *in vitro* ICH model (A) Schematic illustration of the *in vitro* ICH model. (B) TEER values of the *in vitro* ICH model in the presence or absence of fibroblasts. n = 6 biological replicates. ***p = 0.0002 by Student’s t test. (C) Quantification of 4-kDa FITC-dextran leakage in the *in vitro* ICH model in the presence or absence of fibroblasts. n = 6 biological replicates. **p = 0.0016 and **p = 0.0060 for 30- and 60-min time points by Student’s t test, respectively. (D) Representative images of ZO-1 (green), claudin5 (green), and DAPI (blue) in primary human brain microvascular endothelial cells (HBMECs) under normal conditions (uninjured) and at 48 h after *in vitro* ICH with or without fibroblasts. Scale bars, 25 μm. (E) Representative images of caveolin-1 (green), meca32 (red), and DAPI (blue) in primary HBMECs under normal conditions (uninjured) and at 48 h after *in vitro* ICH with or without fibroblasts. Scale bars, 25 μm. (F) Pie chart showing the percentage of each category of proteins relative to total fibroblast-derived proteins identified by LC-MS/MS. (G) TEER values of the *in vitro* ICH model in the presence of mouse IgG (control), PAI1 function-blocking antibody, and TIMP2 function-blocking antibody. n = 6 biological replicates. **p = 0.0088 by Student’s t test. (H) Quantification of 4-kDa FITC-dextran leakage in the *in vitro* ICH model in the presence of mouse IgG (control), PAI1 function-blocking antibody, and TIMP2 function-blocking antibody. n = 6 biological replicates, ***p = 0.00013 and ****p < 0.0001 at 30- and 60-min time points by Student’s t test, respectively. (I) Representative western blot image and quantification of TIMP2 expression secreted by fibroblasts after transduction of lentivirus-expressing TIMP2 short hairpin RNA (shRNA) or a scramble sequence (control). n = 5 biological replicates. *p = 0.0121 by Mann-Whitney U test. (J) TEER values of the *in vitro* ICH model in the presence of control or TIMP2-knockdown fibroblasts. n = 6 biological replicates. **p = 0.0017 by Student’s t test. (K) Quantification of 4-kDa FITC-dextran leakage in the *in vitro* ICH model in the presence of control or TIMP2-knockdown fibroblasts. n = 6 biological replicates. ****p < 0.0001 by Student’s t test. (L) Schematic illustration of *in vitro* TIMP2 rescue experiments. (M) TEER values of the *in vitro* ICH model in the presence of saline (control) or recombinant TIMP2 protein. n = 6 biological replicates. **p = 0.0028 by Student’s t test. (N) Quantification of 4-kDa FITC-dextran leakage in the *in vitro* ICH model in the presence of saline (control) or recombinant TIMP2 protein. n = 6 biological replicates. ***p = 0.0006 and ***p = 0.0007 at 30- and 60-min time points by Student’s t test, respectively. (O) Representative images of ZO-1 (green), claudin5 (green), and DAPI (blue) in primary HBMECs treated with saline (control) or recombinant TIMP2 protein at 48 h after *in vitro* ICH. Scale bars, 25 μm. (P) Representative images of caveolin-1 (green), meca32 (red), and DAPI (blue) in primary HBMECs treated with saline (control) or recombinant TIMP2 protein at 48 h after *in vitro* ICH. Scale bars, 25 μm. Data were shown as mean ± SD. EC, endothelial cell; FB, fibroblast; KD, knockdown. See also Figure S7 and Table S1.

To explore the molecular mechanism underlying fibroblast’s BBB-protective effect, we took advantage of the *in vitro* ICH model established previously^30^ and the Transwell-based *in vitro* BBB model.^31^ Specifically, hemoglobin-activated microglia-conditioned medium was added to the Transwell system containing primary brain microvascular endothelial cells (BMECs) in the upper chamber with or without fibroblasts in the lower chamber (Figure 6A). Compared with the mono-culture system (BMECs alone), the co-culture system (BMECs with fibroblasts) displayed significantly higher trans-endothelial electrical resistance (TEER; Figure 6B) and reduced leakage of 4-kDa fluorescein isothiocyanate (FITC)-dextran (Figure 6C) at 48 h after ICH induction. Similar results were observed when bEnd3 cells (mouse brain endothelial cell line) were used (Figures S7A and S7B). These results suggest that fibroblasts function to enhance BBB integrity in this *in vitro* ICH model.

Consistent with our *in vivo* data, fibroblasts predominantly affected TJP redistribution and expression in the *in vitro* ICH model. In uninjured BMECs, ZO-1 and claudin5 were predominantly found in cell borders (Figure 6D). After *in vitro* ICH, however, this cell border distribution pattern of TJPs was lost (Figure 6D). Interestingly, fibroblasts substantially enhanced the expression of both TJPs at cell borders after ICH (Figure 6D), highlighting an important role of fibroblasts in TJP redistribution. In addition, fibroblasts also dramatically increased total expression levels of ZO-1 (Figure S7C), occludin (Figure S7D), and claudin5 (Figure S7E) in bEnd3 cells after *in vitro* ICH. Unlike TJPs, the expression of transcytosis-related proteins, including caveolin-1 and meca32, was not affected by fibroblasts in BMECs (Figure 6E) or bEnd3 cells (Figure S7F). These results suggest that fibroblasts secrete molecules to repair endothelial barrier integrity after ICH mainly via a paracellular rather than a transcellular mechanism.

### Screening and identification of fibroblast-secreted molecules by mass spectrometry

To screen molecules secreted by fibroblasts, we performed liquid chromatography-tandem mass spectrometry (LC-MS/MS) analysis using concentrated conditioned medium. We identified 66 fibroblast-derived proteins in total (Table S1). Among these proteins, 2 (3.03%) were nuclear proteins, 16 (24.24%) were cytoplasmic proteins, 2 (3.03%) were cell membrane proteins, 25 (37.88%) were ECM proteins, and 21 (31.82%) were secreted proteins (Figure 6F). Given that fibroblasts were able to repair the barrier function without direct contact with endothelial cells (Figures 6A-6E), we mainly focused on secreted proteins. Two such proteins, TIMP2 and PAI1 (plasminogen activator inhibitor 1), were chosen for further studies for two reasons: (1) both proteins were highly expressed by fibroblasts (Table S1), and (2) both proteins showed BBB-protective activity during stroke.^32-35^

### Fibroblast-derived TIMP2 repairs ICH-induced BBB damage *in vitro*

To investigate the functions of TIMP2 and PAI1 in BBB integrity after ICH, we performed loss-of-function studies using function-blocking antibodies in the *in vitro* ICH model. Compared with IgG control, TIMP2 function-blocking antibody significantly decreased TEER (Figure 6G) and increased 4-kDa FITC-dextran leakage (Figure 6H) in the endothelium-fibroblast co-culture system. Interestingly, PAI1 function-blocking antibody failed to affect TEER (Figure 6G) or 4-kDa FITC-dextran leakage (Figure 6H). These results suggest that TIMP2, rather than PAI1, contributes to endothelial barrier integrity in the *in vitro* ICH model. To further validate the function of fibroblast-derived TIMP2 in BBB integrity, we knocked down TIMP2 expression in fibroblasts using the lentivirus-mediated RNAi technique (Figure 6I). Consistent with the pharmacological approach, knockdown of TIMP2 in fibroblasts substantially lowered TEER (Figure 6J) and enhanced 4-kDa FITC-dextran leakage (Figure 6K) compared with the control. Together, these results suggest that fibroblasts repair BBB damage after ICH at least partially via TIMP2.

### TIMP2 repairs ICH-induced BBB damage

To determine whether TIMP2 can reverse ICH-induced BBB damage, we first performed an *in vitro* rescue experiment using primary BMECs (Figure 6L). Recombinant TIMP2 substantially enhanced TEER (Figure 6M) and reduced 4-kDa FITC-dextran leakage (Figure 6N) in the *in vitro* ICH model. In addition, TIMP2 increased the expression of ZO-1 and claudin5 at cell borders in BMECs (Figure 6O) without affecting that of caveolin-1 or meca32 (Figure 6P). These findings suggest that TIMP2 attenuates *in vitro* ICH-induced vascular leakage mainly through the paracellular mechanism.

**Figure 7.**
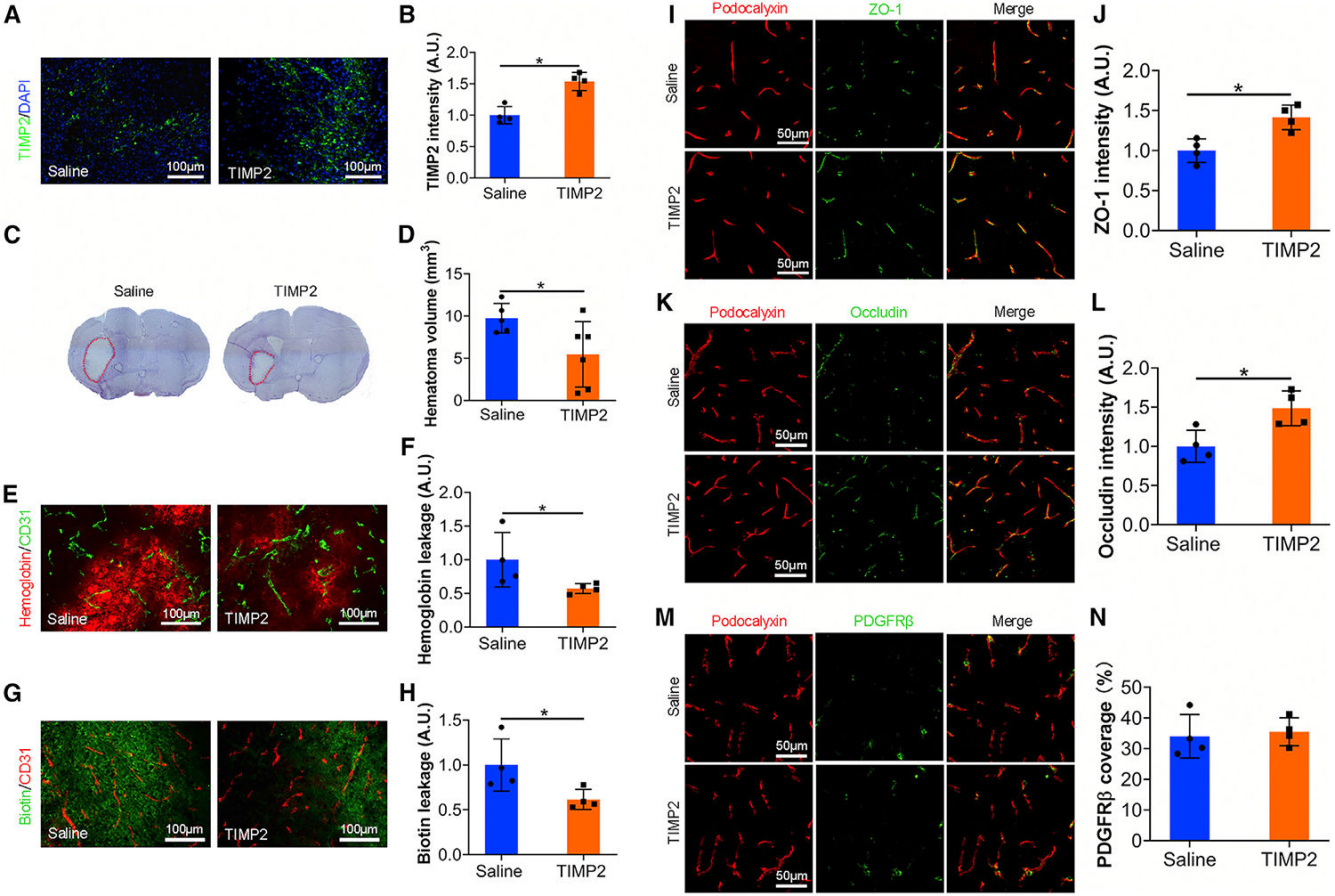
TIMP2 treatment enhances BBB integrity in FKO mice *in vivo* (A) Representative images of TIMP2 (green) and DAPI (blue) in the peri-hematoma regions of saline- and TIMP2-treated FKO mice at day 7 after ICH. Scale bars, 100 μm. (B) Quantification of TIMP2 intensity in the peri-hematoma regions of saline- and TIMP2-treated mice at day 7 after ICH. n = 4 mice. *p = 0.0286 by Mann-Whitney U test. (C) Representative images of cresyl violet staining in saline- and TIMP2-treated FKO mice at day 7 after ICH. Dotted lines indicate injury areas. (D) Quantification of injury volume in saline- and TIMP2-treated mice at day 7 after ICH. n = 5–6 mice. *p = 0.0491 by Student’s t test. (E) Representative images of hemoglobin (red) and CD31 (green) in the peri-hematoma regions of saline- and TIMP2-treated mice at day 7 after ICH. Scale bars, 100 μm. (F) Quantification of hemoglobin intensity in the peri-hematoma regions of saline- and TIMP2-treated mice at day 7 after ICH. n = 4 mice. *p = 0.0286 by Mann-Whitney U test. (G) Representative images of biotin (green) and CD31 (red) in the peri-hematoma regions of saline- and FKO-treated mice at day 7 after ICH. Scale bars, 100 μm. (H) Quantification of biotin intensity in the peri-hematoma regions of saline- and TIMP2-treated mice. n = 4 mice. *p = 0.0286 by Mann-Whitney U test. (I) Representative images of ZO-1 (green) and podocalyxin (red) in the peri-hematoma regions of saline- and TIMP2-treated mice at day 7 after ICH. Scale bars, 50 μm. (J) Quantification of ZO-1 intensity normalized to podocalyxin-positive area in saline- and TIMP2-treated mice. n = 4 mice. *p = 0.0286 by Mann-Whitney U test. (K) Representative images of occludin (green) and podocalyxin (red) in the peri-hematoma regions of saline- and TIMP2-treated mice at day 7 after ICH. Scale bars, 50 μm. (L) Quantification of occludin intensity normalized to podocalyxin-positive area in saline- and TIMP2-treated mice. n = 4 mice. *p = 0.0286 by Mann-Whitney U test. (M) Representative images of PDGFRβ (green) and podocalyxin (red) in the peri-hematoma regions of saline- and TIMP2-treated mice at day 7 after ICH. Scale bars, 50 μm. (N) Quantification of PDGFRβ coverage over podocalyxin-positive capillaries in saline- and TIMP2-treated mice. n = 4 mice. Data were shown as mean ± SD.

Next, we further investigated the effects of TIMP2 in BBB integrity and ICH outcome *in vivo*. Recombinant TIMP2 or saline (control) was infused into the brains of FKO mice for 4 days (days 3–7 after ICH) using osmotic pumps. Immunohistochemistry showed that this approach substantially elevated TIMP2 level in ICH brains (Figures 7A and 7B). TIMP2 treatment substantially decreased hematoma volume in FKO mice at day 7 after ICH (Figures 7C and 7D). In addition, TIMP2-treated FKO mice showed significantly reduced accumulation of hemoglobin (Figures 7E and 7F) and biotin (Figures 7G and 7H) in brain parenchyma compared with the controls, indicating improved BBB integrity. Consistent with our mechanistic data, ZO-1 (Figures 7I and 7J) and occludin (Figures 7K and 7L) levels were dramatically up-regulated in TIMP2-treated FKO mice. These results suggest that TIMP2 repairs ICH-induced BBB damage *in vivo*. Interestingly, PDGFRβ coverage was not rescued by TIMP2 treatment in FKO mice (Figures 7M and 7N), indicating a minimal role of TIMP2 in pericyte coverage in this model. Together, these findings suggest that Col1α1^+^ fibroblasts repair BBB damage and hemorrhagic brain injury partially via TIMP2.

## DISCUSSION

Our lineage-tracing study showed that Col1α1^+^ cells were mainly located in the meninges and large blood vessels in the brain under homeostatic conditions, and that these Col1α1^+^ cells expressed all fibroblast markers examined, including Col1, PDGFRα, and RALDH2, although only partial co-localization was observed. These anatomical and biochemical features suggest that Col1α1-Cre specifically labels a subpopulation of fibroblasts in the CNS. It is worth noting that the activity of the *Col1α1* promoter is weak under homeostatic conditions because only ~35% of Col1^+^ cells express tdTomato. Consistent with the highly responsive nature of fibroblasts to injuries, Col1α1^+^ cells increased dramatically after ICH and accumulated predominantly in the peri-hematoma area in the subacute phase, indicating that Col1α1^+^ fibroblasts may proliferate and contribute to the formation of fibrotic scar in ICH. Interestingly, proliferation of large blood vessel-derived PDGFRβ^+^CD105^+^ stromal cells, which are probably fibroblasts, was also observed in both mice and humans at the subacute stage after ischemic stroke.^36^ Consistent with this observation, fibroblasts have been shown to contribute to the formation of fibrotic scar after ischemic stroke.^8^ In addition, there is also evidence supporting that fibroblasts become activated and contribute to fibrotic scar formation in other neurological disorders, including SCI and EAE.^4^ These results suggest that fibroblast proliferation/activation and fibrotic scar formation may be common changes after CNS injury.

Col1α1^+^ cells were predominantly found in the fibrotic scar and the region immediately outside the scar in the subacute phase after ICH. In the fibrotic scar, Col1α1^+^ cells expressed a variety of fibroblast markers and clustered together, highlighting a fibroblast nature. In the region immediately outside the scar, however, Col1α1^+^ cells lost fibroblast marker Col1, co-localized with mural cell marker PDGFRβ, and were associated with small blood vessels, suggesting that these cells may have pericyte-like properties. In support of this speculation, mice with ablated Col1α1^+^ fibroblasts exhibited decreased pericyte coverage after ICH. In addition, as a vessel-associated cell type in the CNS, fibroblasts promote vessel stabilization during development.^37^ Together, these results suggest that Col1α1^+^ fibroblasts may migrate to capillaries to modulate BBB integrity and ICH outcome.

Using mice with ablated Col1α1^+^ fibroblasts, we demonstrated that Col1α1^+^ fibroblasts exerted a beneficial role in ICH by promoting BBB repair. In accordance with our findings, a recent study reported that PDGFRα/Col1-expressing fibroblasts protected against BBB dysfunction and inhibited hemorrhagic transformation in the subacute phase of ischemic stroke.^13^ These results suggest that fibroblasts play an essential role in BBB repair after stroke. Consistent with the beneficial role of fibroblasts, reducing GLAST- and PDGFRβ-expressing “type A” pericytes, which likely contain fibroblasts, leads to unsealed injury in an SCI model.^14^ It should be noted, however, that a detrimental role of fibroblasts has also been reported. For instance, fibroblasts inhibit neuronal regeneration and functional recovery in the remodeling stage of SCI by secreting an excessive amount of ECM proteins.^38^ Similarly, ablation of proliferating Col1α2-expressing cells leads to increased oligodendrocyte lineage cells and improved motor function in the chronic stage of EAE.^7^ This disparity may be explained by different fibroblast markers used in these studies. Fibroblasts are a heterogeneous population with many subpopulations.^1,2^ It is likely that Col1α1, Col1α2, GLAST, PDGFRα, and PDGFRβ label different subpopulations of fibroblasts, which have distinct functions. Understanding the function of each individual fibroblast subpopulation will significantly enrich our knowledge in fibroblast biology. Techniques, such as single-cell RNA-seq analysis, may be used to identify markers and properties of different fibroblast subpopulations. It should be noted that none of the above-mentioned markers, except Col1α1, are fibroblast specific. They also label other cell types that may affect disease outcome. In addition, different diseases and disease stages may also contribute to this discrepancy. It is possible that fibroblasts exert distinct functions in different injury models and/or at different stages after injury. For instance, fibroblasts may actively regulate BBB integrity in the subacute phase, while the dense fibrotic scar may prevent neurons and other cells from penetrating the injured tissue at the remodeling phase. Therefore, it is critical to study fibroblast functions at multiple stages.

BBB disruption is a key pathology of ICH and correlates with stroke outcome.^22,39^ In this study, we found that Col1α1^+^ fibroblasts promote BBB repair in a TIMP2-dependent manner. This is consistent with previous studies showing that TIMP2 protects against BBB disruption in both ischemic and hemorrhagic stroke.^32,33,40,41^ TIMP2 is an endogenous inhibitor of matrix metalloproteinase-2 (MMP-2), an MMP that actively regulates ECM turnover/remodeling and BBB integrity in physiological and pathological conditions.^41,42^ Because MMP-2 is substantially up-regulated in stroke^43,44^ and increased MMP-2 induces BBB breakdown,^32,33,40,41^ it is believed that TIMP2 exerts its neuroprotective role in stroke via inhibiting MMP-2. It should be noted, however, that TIMP2 may also function in an MMP-independent manner. It has been shown that TIMP2 decreases vascular permeability in tumors via MMP-independent mechanisms.^45^ Whether TIMP2 promotes BBB repair in ICH through MMP-2 inhibition or an MMP-independent manner needs further study.

We found that TIMP2 treatment attenuated BBB disruption and up-regulated TJPs in Col1α1^+^ fibroblast-ablated mice but failed to affect pericyte coverage, suggesting a minimal role of TIMP2 in pericyte coverage after ICH. This partial rescue suggests that other factors also contribute to the phenotype in Col1α1^+^ fibroblast-ablated mice after ICH. As a major cellular source of ECM proteins, growth factors, and inflammatory cytokines, fibroblasts may regulate BBB integrity and stroke outcome through these mechanisms. Characterizing the expression profile of fibroblasts in ICH will provide insights on the molecular mechanisms of fibroblast-mediated ICH recovery.

PAI1, an endogenous inhibitor of tissue-type plasminogen activator, was also identified as a fibroblast-secreted factor that may regulate BBB integrity in the LC-MS/MS study. However, loss-of-function studies demonstrated that PAI1, unlike TIMP2, failed to affect BBB permeability in our *in vitro* ICH model. This is in contrast with previous findings that PAI1 protects BBB integrity and inhibits hemorrhagic transformation in ischemic stroke.^34,46^ This discrepancy may be explained by different experimental models/conditions. It is possible that tissue-type plasminogen activator level in this simplified *in vitro* ICH model is not high enough to induce functional changes when PAI1 is abrogated. Therefore, the expression of tissue-type plasminogen activator and the functional significance of PAI1 in ICH should be assessed *in vivo* in further research.

In this study, we report that Col1α1^+^ fibroblasts repair BBB damage after ICH partially via TIMP2. It is possible that Col1α1^+^ fibroblasts are involved in BBB recovery in other neurological disorders. A recent single-cell RNA-seq study showed that perivascular fibroblasts were significantly under-represented in the cortex of Alzheimer disease patients.^47^ The reduced fibroblast population may affect TIMP2 and MMP-2 levels and/or activities, which have been shown to regulate BBB integrity and Alzheimer disease pathology.^48^ In addition, Col1α1^+^ fibroblasts may repair BBB injury in multiple sclerosis and traumatic brain injury using a similar mechanism. It has been reported that these diseases share common endothelial gene expression changes known as the core BBB dysfunction module, which includes many ECM genes and modulators of the ECM (e.g., extracellular proteases and protease inhibitors).^49^

### Limitations of the study

Although the Col1α1-Cre line specifically labels fibroblasts, its promoter activity is too low under homeostatic conditions. We failed to induce fibroblast depletion in homeostatic adult animals. After ICH injury, however, *Col1α1* promoter activity was substantially up-regulated, which successfully induced fibroblast depletion. Thus, the Col1α1-Cre line is insufficient for fibroblast depletion under homeostatic conditions but can be used to specifically target Col1α1^+^ fibroblasts after ICH or possibly other injuries that activate fibroblasts.

In addition, it should be noted that the Col1α1-Cre line labels only a subpopulation of fibroblasts (Col1α1^+^ fibroblasts), and that it does not distinguish meningeal fibroblasts and perivascular fibroblasts. Therefore, it remains unclear which population exerts the neuroprotective function in ICH. Given that perivascular fibroblasts are much closer to the hematoma in the striatum than meningeal fibroblasts, we hypothesize that perivascular fibroblasts rather than meningeal fibroblasts repair BBB damage and hemorrhagic brain injury via TIMP2. This hypothesis, however, needs to be tested in the future using tools that specifically mark perivascular fibroblasts.

## STAR★METHODS

### RESOURCE AVAILABILITY

#### Lead contact

Further information and requests for resources and reagents should be directed to and will be fulfilled by the lead contact, Yao Yao (yao7@usf.edu).

#### Materials availability

Materials will be shared upon request within the limits of the respective material transfer agreements.

#### Data and code availability

- The mass spectrometry proteomics data have been deposited to the ProteomeXchange Consortium via the PRIDE partner repository with the dataset identifier Database: PXD037247 and are publicly available as of the date of publication.
- This paper does not report original code.
- Any additional information required to reanalyze the data reported in this paper is available from the lead contact upon request.

### EXPERIMENTAL MODEL AND SUBJECT DETAILS

#### Mouse generation

For fibroblast ablation experiment, the Col1α1-Cre^+^ mice were crossed with the Cre-inducible diphtheria toxin receptor (DTR) mice^50^ to generate DTR^+/−^:Col1α1-Cre^+^ (termed FKO) mice. Littermate DTR^+/−^ mice with equal amount of diphtheria toxin (DT, sigma-Aldrich) injection were used as controls for FKO mice. For lineage-tracing experiment, the Ai14 reporter line was crossed with Col1α1-Cre^+^ mice to generate Ai14^+/−^:Col1α1-Cre^+^ (Col1α1-tdTomato) mice, in which Col1α1^+^ cells and their progenies were permanently labeled with tdTomato. In these experiments, mice of both genders at ~2 months old were used.

#### Mouse maintenance

All mice were maintained in the animal facility at the University of South Florida. They were kept in ventilated cages with free access to water and food, under specific pathogen-free conditions and 12 h/12 h light/dark cycle. All procedures were in compliance with the NIH guide and approved by the Institutional Animal Care and Use Committee (IACUC).

### METHOD DETAILS

#### Diphtheria toxin injection

For homeostatic studies, diphtheria toxin (DT, sigma-Aldrich) was administered into control and FKO mice either intraperitoneally or intraventricularly. For the former, 500ng DT was injected daily for 5 days continuously and mice were analyzed 24 h after the last injection. For the latter, 1500ng DT was directly delivered into the lateral ventricle over 5 days using Alzet Osmotic Pumps (model #1002) and the Brain Infusion Kit. For ICH studies, FKO mice were injected with 500ng DT daily for 6 days continuously and then every other day until sacrifice. ICH was induced on the third day of DT injection and mice were analyzed at various days after ICH. DT injection strategies were presented in Figures 2A and S1A.

#### ICH model

ICH was induced as described in our previous publications.^51,52^ Briefly, mice were anesthetized via intraperitoneal injection of avertin (500 mg/kg of body weight), and secured on the stereotaxic instrument (Stoelting Co., IL, USA). A bur hole was drilled on the skull at the following coordinates: 0.2 mm posterior to bregma, 2.4 mm lateral from the midline, and 3.7 mm in depth. Collagenase (type VIIS; Sigma, St. Louis, USA; 0.15U in 0.5μL saline) was then injected into the right striatum using a 30-gauge syringe (Hamilton) maneuvered by the stereotaxic instrument. The needle was kept in place for 5 min to avoid reflux after injection.

#### *In vivo* BBB permeability assay

BBB permeability was assessed *in vivo* as described in our previous publications.^53^ Briefly, 50ul of 5mg/ml Sulfo-NHS-Biotin (ThermoFisher, 21217) in sterile saline was injected into control and FKO mice intravenously. After 6 h of circulation, mice were transcardially perfused with 4% PFA. In brain sections, biotin was detected and visualized with Avidin-FITC (1:200, BD, 554057). Mouse IgG and hemoglobin were co-immunostained with vessel marker CD31. The mean fluorescent intensity of biotin, IgG and hemoglobin was determined using at least three images per section, four-eight sections evenly distributed along the hematoma, and at least four mice using ImageJ software.

#### *In vivo* TIMP2 treatment

Alzet Osmotic Pumps (model #1002) and the Brain Infusion Kit were implanted into FKO mice at day 3 after ICH induction according to manufacturer instructions. Briefly, mice were anesthetized with Avertin (500 mg/kg of body weight) and treated with carprofen (5mg/kg of body weight) subcutaneously. Cannulas were implanted into stroke area. A total of 0.8 μg recombinant human TIMP2 (R&D, 971-TM-010) in sterile saline was delivered into each mouse over 4 days. Osmotic pumps filled with sterile saline were used as controls. Brain samples were collected at day 7 after ICH.

#### Neurological deficit

Mice were scored for neurological deficits at various time points after ICH using a modified scoring system.^54,55^ In this system, six properties, including body symmetry, gait, climbing, circling behavior, front limb symmetry, and compulsory circling, were graded from 0 to 4, with a maximum score of 24. Higher score indicates more severe neurological deficits. The genotypes of mice were with-held from researchers, who performed this test.

#### Immunohistochemistry

Immunohistochemistry was performed as described in our previous publications.^56^ Briefly, brain sections were fixed in cold 3% glyoxal for 30 min or 4% PFA for 20 min. After extensive washes with PBS, the sections were incubated in blocking buffer (1% BSA in PBS containing 0.3% normal donkey serum and 0.3% Triton X-100) for 1 h at room temperature. Next, the sections were incubated with primary antibodies overnight at 4°C. After extensive washes in PBS, the sections were incubated with appropriate secondary antibodies for 1 h at room temperature. Then, the sections were washed in PBS for 3 times and mounted with Fluoromount-G with DAPI. Nikon Eclipse TiE microscope and LSM710 confocal microscope were used to take images, which were further processed by ImageJ and/or Adobe Photoshop. For ICH brains, images were taken from the peri-hematoma regions.

#### Transmission electron microscopy

Mice were anesthetized and perfused with PBS followed by 0.1M sodium cacodylate buffer containing 2% PFA and 2% glutaraldehyde. Brain tissue from the perihematomal zone was dissected out, fixed overnight, and post-fixed in 1% osmium tetroxide and 1% potassium ferrocyanide. Next, the collected brain tissue was *en bloc* stained with 2% uranyl acetate and embedded in resin. An RMC MT-X microtome (Boeckeler Instruments) was used to cut ultra-thin sections, which were post-stained with 2% uranyl acetate and 1% lead citrate. Sections were examined and photographed using JEOL JEM1011 (JEOL) at 80 kV.

#### Cell culture

Mouse brain endothelial cells (bEnd.3, CRL-2299), mouse bone marrow cells (LADMAC, CRL-2420), and mouse brain microglia (EOC 13.31, CRL-2468) were purchased from ATCC. Human brain microvascular endothelial cells (HBMEC, 1000) and human brain vascular adventitial fibroblasts (HBVAF, 1110) were purchased from ScienCell. bEnd.3 and LADMAC cells were cultured in standard medium [Dulbecco’s modified Eagle’s medium (DMEM) supplemented with 10% fetal bovine serum (FBS), 100 units/ml penicillin and 100 μg/mL streptomycin]. EOC 13.31 cells were cultured in standard medium supplemented with 20% LADMAC conditioned media. HBMEC cells were cultured in Endothelial Cell Medium (ECM, Cat. #1001, ScienCell) and HBVAF cells were cultured in Fibroblast Medium (FM, Cat. #2301, ScienCell). All cells were cultured at 37°C with 5% CO_2_ atmosphere.

#### *In vitro* ICH model

The *in vitro* ICH model was established as described previously.^30^ Briefly, EOC 13.31 microglia cells were treated with 10μM hemoglobin (Sigma, H7379) for 48 h and the supernatant was collected as microglia-conditioned medium. Next, 2 × 10^4^ HBMEC or bEnd3 cells were seeded into Transwell inserts (Corning Costar, 3472). When the cells reached confluence, fibroblasts were seeded in the lower chamber. The next day, microglia-conditioned medium was added to both the upper and lower chambers to mimic ICH *in vitro*.

#### *In vitro* BBB permeability assays

*In vitro* BBB permeability was examined by assessing trans-endothelial electrical resistance (TEER) and tracer leakage as described previously.^31,57-59^ For the former, TEER was measured using an Epithelial-volt-ohm-meter (EVOM, World Precision Instruments Inc.). For the latter, 100μg/ml 4kD-FITC dextran (sigma, 46,944) was added to the upper chamber and its leakage into the lower chamber was determined using a fluorescent plate reader.

#### TIMP2 and PAI1 blockage

To functionally block TIMP2 and PAI1 secreted by fibroblasts, 5μg/ml TIMP2 blocking antibody (R&D, AF971) and 2.5μg/ml PAI1 blocking antibody (R&D, MAB1786) were added to the lower chamber in the *in vitro* ICH system, respectively. 5μg/ml mouse IgG (R&D, MAB002) was used as a control.

#### *In vitro* TIMP2 treatment

*In vitro* rescue experiment was performed using primary HBMEC cells. Briefly, HBMEC cells were grown in Transwell inserts (Corning Costar, 3472) and *in vitro* ICH model was induced in the presence of 10nM recombinant human TIMP2 protein (R&D, 971-TM-010) or saline as described above. HBMEC barrier function was determined using *in vitro* BBB permeability assays.

#### TIMP2 knockdown

RNAi technique was used to knock down TIMP2 expression in fibroblasts. Briefly, lentivirus expressing shRNA targeting TIMP2 (sc-29506-V) or a scramble sequence (control, sc-108080) was used to transduce fibroblasts, according to manufacturer’s instructions. Briefly, fibroblasts were plated in 12-well plates. When they reached 50% confluence, complete medium with 5μg/ml polybrene and lentivirus (MOI = 1) was added. The medium was replaced 12 h after transduction. Complete medium with 2μg/ml puromycin (Gibco, A1113803) was used to select clones expressing shRNA 48 h after transduction.

#### LC-MS/MS analysis

FBS-free medium was used in LC-MS/MS analysis. Specifically, mouse microglia were cultured in FBS-free DMEM containing 5μM hemoglobin for 24 h. The resulting microglia-conditioned medium was used to treat human fibroblasts for 24 h to collect fibroblast-conditioned medium. The microglia- and fibroblast-conditioned media were concentrated using Amicon Ultra centrifuge filters (MWCO 10 kDa, Millipore, Z677108), and proteins were separated in gradient SDS-PADE. Hemoglobin bands were cut off from the gel and the remaining gel pieces were pooled and submitted to the Proteomics and Mass Spectrometry Facility for LC-MS/MS analysis. Briefly, proteins were digested using an in-gel trypsin digestion protocol. Mass spectrometry analyses were performed on a Thermo-Fisher LTQ Orbitrap Elite Mass Spectrometer coupled with a Proxeon Easy NanoLC system (Waltham, MA). Data were acquired using Xcalibur software (version 2.2, Thermo Fisher Scientific). Protein identification and modification characterization were performed using Thermo Proteome Discoverer (version 1.4) with Mascot (Matrix Science 2.7) and UniProt database. The spectra of possible modified peptides were inspected further to verify the accuracy of the assignments.

#### RNAScope *in situ* hybridization

*In situ* hybridization was conducted using the RNAscope multiplex fluorescent reagent kit V2 (Advanced Cell Diagnostics, 323,100), according to the manufacturer’s instructions. *Col1α1*-specific oligo probe (319,371-C3) and Opal^™^ dyes were used to visualize *Col1α1* mRNA expression. Sections were imaged using an Olympus BX53 fluorescent microscope and analyzed using NIH ImageJ software.

#### Western blotting

Cells were lysed with RIPA buffer (50-mM Tris pH 7.4, 1% NP-40, 0.5% Na-deoxycholate, 1% SDS, 150-mM NaCl, 2-mM EDTA, 1 x protease inhibitor cocktail, and 1 x phosphatase inhibitor cocktail). Total protein levels were determined using the Bio-Rad protein assay kit, and equal amounts of proteins were loaded and separated on SDS-PAGE. After transferring to PVDF membrane (Millipore), proteins of interest were detected using a standard immune-blotting technique. The following primary antibodies were used: mouse anti-claudin-5 (1:500, Invitrogen, 35–2500), rabbit anti-ZO-1 (1:500, Thermofisher, 61–7300), rabbit anti-caveolin-1 (1:1000, cell signaling, 3238S), rat anti-meca32 (1:200, Novus, NB100-77668), goat anti-TIMP2 (1:200, R&D, AF971), and mouse anti-actin (1:2000, Sigma, A5441). SuperSignal West Pico Plus Chemiluminescent Substrate (Thermo scientific) was used to detect protein bands. NIH ImageJ software was used to quantify the density of target protein bands. The expression of target proteins was normalized to that of β-actin.

### QUANTIFICATION AND STATISTICAL ANALYSIS

#### Image analyses

Brain injury was revealed by cresyl violet staining and injury volume was quantified on serial sections using the NIS-Elements D3.0 software as described previously.^52,54^ Briefly, hematoma area (mm^2^) from serial sections were added, and the injury volume (mm^3^) was calculated as measured area x distance between sections. Degenerating neurons were visualized and evaluated by Fluoro-Jade C (FJC) staining as described previously.^54,60^ FJC^+^ cells were quantified using three fields immediately adjacent to the hematoma per section, four-eight sections evenly distributed along the hematoma, and at least four mice. The numbers of degenerating cells were shown as number of cells per field. All data analyses were performed by a blinded investigator.

Col1α1-Cre labeling efficiency was quantified as the percentage of tdTomato fluorescent area (or length) over Col1 fluorescent area (or length) in Col1α1-tdTomato brains under homeostatic conditions. PDGFRβ coverage, CD13 coverage, and AQP4 coverage were defined as the percentages of PDGFRβ, CD13, and AQP4 fluorescent areas covering CD31-or podocalyxin-positive capillary area, respectively, as described previously.^61^ ZO-1/occludin intensity, PDGFRβ/CD13 intensity, and AQP4 intensity were defined as integrated fluorescence intensity normalized by CD31^+^ or podocalyxin^+^ capillary area, as described previously.^52^ For hemoglobin/IgG/sulfo-biotin leakage and Col1/meca32 expression, mean fluorescence intensity was used. PDGFRα^+^Olig2^−^ fibroblasts were determined by counting DAPI^+^ nuclei within PDGFRα^+^Olig2^−^ areas. Endothelial caveolin-1 level was quantified as the mean gray value of caveolin-1 signal within blood vessels. Briefly, podocalyxin^+^ blood vessels were outlined, set as ROIs, and overlaid in the corresponding caveolin-1 images. Then the mean gray value of caveolin-1 signal within the set ROIs was measured using ImageJ (NIH). For *in situ* hybridization, *Col1α1* (mRNA)-expressing cells were determined by counting the number of DAPI^+^ nuclei surrounded by *Col1α1* signal. For quantifications, at least three fields randomly selected from each section, four-eight sections evenly distributed along the hematoma or rostral-caudal axis of the brain, and at least four mice were used. All data analyses were performed by a blinded investigator.

Endothelial transcytosis was measured using transmission electron microscopy images as described previously.^62^ Briefly, endothelial pinocytotic vesicles in control and FKO mice at day 7 after ICH were manually counted and normalized to endothelial area. Twenty-five to twenty-seven capillaries from three mice were used for quantification and data analysis was performed by a blinded investigator.

#### Statistical analyses

Statistical analyses were conducted using Prism 8 (GraphPad Software). Student’s t test and/or Mann-Whitney U test were used to examine differences between two independent groups. One-way ANOVA followed by Neuman Keuls post hoc analysis was applied when more than two groups were compared. p < 0.05 was considered as significant. Results were shown as mean ± SD.

## Supplementary Material

1

2

3

4

5

## Figures and Tables

**Table:** KEY RESOURCES TABLE

REAGENT or RESOURCE	SOURCE	IDENTIFIER
Antibodies		
Rat anti-CD31	BD Biosciences	Cat#553370; RRID:AB_394816
Goat anti-Podocalyxin	R&D	Cat#AF1556; RRID:AB_354858
Rabbit anti-RALDH2	Sigma	Cat#HPA010022; RRID:AB_1844723
Mouse anti-NG2	BD Biosciences	Cat#554275; RRID:AB_395339
Mouse anti-Desmin	Millipore	Cat#IF02L; RRID:AB_2261688
Rabbit anti-Olig2	Novus	Cat#NBP1-28667; RRID:AB_1914109
Rabbit anti-ZO-1	Innovative Research	Cat#61-7300; RRID:AB_138452
Rabbit anti-AQP4	Millipore	Cat#AB3594; RRID:AB_91530
Rabbit anti-Col1	Millipore	Cat#AB765P; RRID:AB_92259
Rabbit anti-Occludin	Invitrogen	Cat#71-1500; RRID:AB_2533977
Mouse anti-Claudin5	Invitrogen	Cat#35-2500; RRID:AB_2533200
Rat anti-Meca32	Novus	Cat#NB100-77668; RRID:AB_2276108
Rabbit anti-Caveolin-1	Cell Signaling	Cat#3238; RRID:AB_2072166
Rabbit anti-PDGFRβ	Cell Signaling	Cat#3169; RRID:AB_2162497
Goat anti-PDGFRα	R&D	Cat#AF1062; RRID:AB_2236897
Goat anti-TIMP2	R&D	Cat#AF971; RRID:AB_355752
Mouse anti-PAI1	R&D	Cat#MAB1786; RRID:AB_2186903
Mouse IgG	R&D	Cat#MAB002; RRID:AB_357344
Rat anti-CD13-FITC	BD Biosciences	Cat#558744; RRID:AB_397101
Rabbit anti-Hemoglobin	Cloud-Clone	Cat#PAB409Mu01
Mouse anti-Actin	Sigma	Cat#A5441; RRID:AB_476744
Alexa Fluor-405 conjugated donkey anti-rat	Invitrogen	Cat#A48268; RRID:AB_2890549
Alexa Fluor-488 conjugated donkey antirabbit	Invitrogen	Cat#A21206; RRID:AB_2535792
Alexa Fluor-594 conjugated donkey antirabbit	Invitrogen	Cat#A21207; RRID:AB_141637
Alexa Fluor-594 conjugated donkey anti-mouse	Invitrogen	Cat#A21203; RRID:AB_141633
Alexa Fluor-594 conjugated donkey anti-rat	Invitrogen	Cat#A21209; RRID:AB_2535795
Alexa Fluor-647 conjugated goat anti-rat	Invitrogen	Cat#A21247; RRID:AB_141778
FITC conjugated goat anti-mouse	BD Pharmingen	Cat#554001; RRID:AB_395197
FITC conjugated goat anti-rat	BD Pharmingen	Cat#554016; RRID:AB_395210
Bacterial and virus strains		
Lentivirus-siRNA-TIMP2	Santa Cruz	Cat#SC-29506
Lentivirus-siRNA-Control	Santa Cruz	Cat#SC-108080
Chemicals, peptides, and recombinant proteins		
Diphtheria Toxin	Sigma-Aldrich	Cat#D0564
Sulfo-NHS-Biotin	ThermoFisher	Cat#21217
Avidin-FITC	BD Biosciences	Cat#554057
Recombinant Human TIMP-2 Protein	R&D	Cat#971-TM-010
Critical commercial assays		
RNAScope *in situ* hybridization	Advanced Cell Diagnostics	Cat#323100
Deposited data		
Raw and processed MS data	ProteomeXchange Consortium	Database: PXD037247
Experimental models: Cell lines		
Mouse brain endothelial cells (bEnd.3)	ATCC	Cat#CRL-2299; RRID:CVCL_0170
Mouse bone marrow cells (LADMAC)	ATCC	Cat#CRL-2420; RRID:CVCL_2550
Mouse brain microglia (EOC 13.31)	ATCC	Cat#CRL-2468; RRID:CVCL_5743
Human brain microvascular endothelial cells (HBMEC)	ScienCell	Cat#1000
Human brain vascular adventitial fibroblasts (HBVAF)	ScienCell	Cat#1110
Experimental models: Organisms/strains		
Mouse: B6.FVB-Tg(Col1a1-cre)1Kry	Riken BRC	BRC: RBRC05603; RRID:IMSR_RBRC05603
Mouse: B6.Cg-Gt(ROSA)26Sor^tm14(CAG-tdTomato)Hze^/J	The Jackson Laboratory	JAX:007914; RRID:IMSR_JAX:007914
Mouse: C57BL/6-Gt(ROSA)26Sor*^tm1(HBEGF)Awai^*/J	The Jackson Laboratory	JAX:007900; RRID:IMSR_JAX:007900
Software and algorithms		
ImageJ	NIH	https://imagej.nih.gov/ij/
Prism 8	GraphPad	https://www.graphpad.com/scientific-software/prism/
Photoshop	Adobe	N/A

## Highlights

- Loss of Col1α1^+^ fibroblasts exacerbates hemorrhagic brain injury
- Ablation of Col1α1^+^ fibroblasts aggravates BBB damage after ICH
- Col1α1^+^ fibroblasts repair ICH-induced BBB damage via paracellular mechanism
- Col1α1^+^ fibroblasts repair ICH-induced BBB damage in a TIMP2-dependent manner
