# Supplementary material for: Fibroblasts repair blood-brain barrier damage and hemorrhagic brain injury via TIMP2
Source: Cell Rep. Author manuscript; Available in PMC 2022 Dec 21. (PMC9769996; doi:10.1016/j.celrep.2022.111709)

Cell Reports, Volume *41*

Supplemental information

Fibroblasts repair blood-brain barrier damage and hemorrhagic brain injury via TIMP2

Lingling Xu, Abhijit Nirwane, Ting Xu, Minkyung Kang, Karan Devasani, and Yao Yao

SUPPLEMENTAL FIGURES

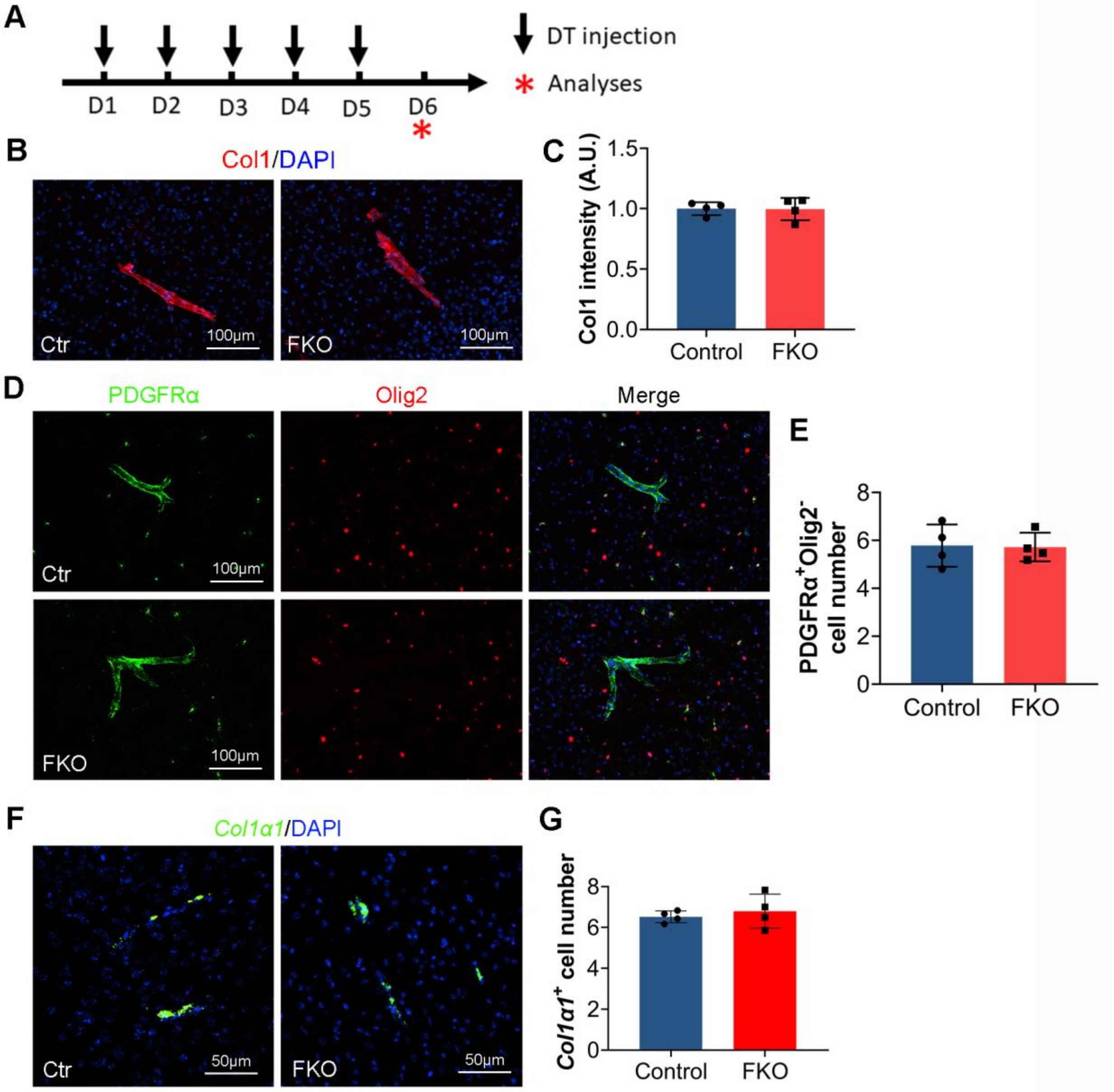

Figure S1. Col1α1$^+$ fibroblasts are not ablated in FKO mice under homeostatic conditions with intraperitoneal injection of DT.

(A) Schematic illustration of time points for DT injection.

(B) Representative images of Col1 (red) and DAPI (blue) in control and FKO mice under homeostatic conditions with intraperitoneal injection of DT. Scale bars = 100μm.

(C) Quantification of Col1 intensity in control and FKO brains. n = 4 mice.

(D) Representative images of PDGFRα (green), Olig2 (red), and DAPI (blue) in control and FKO mice under homeostatic conditions with intraperitoneal injection of DT. Scale bars = 100μm.

(E) Quantification of PDGFRα$^+$Olig2$^-$ cells in control and FKO brains. n = 4 mice.

(F) Representative images of *Col1α1* mRNA (green, detected by RNAscope in situ hybridization) and DAPI (blue) in control and FKO mice under homeostatic conditions with intraperitoneal injection of DT. Scale bars = 50μm.

(G) Quantification of *Col1α1*$^+$ cells in control and FKO brains. n = 4 mice.

Data were shown as mean ± SD. Related to Figure 1.

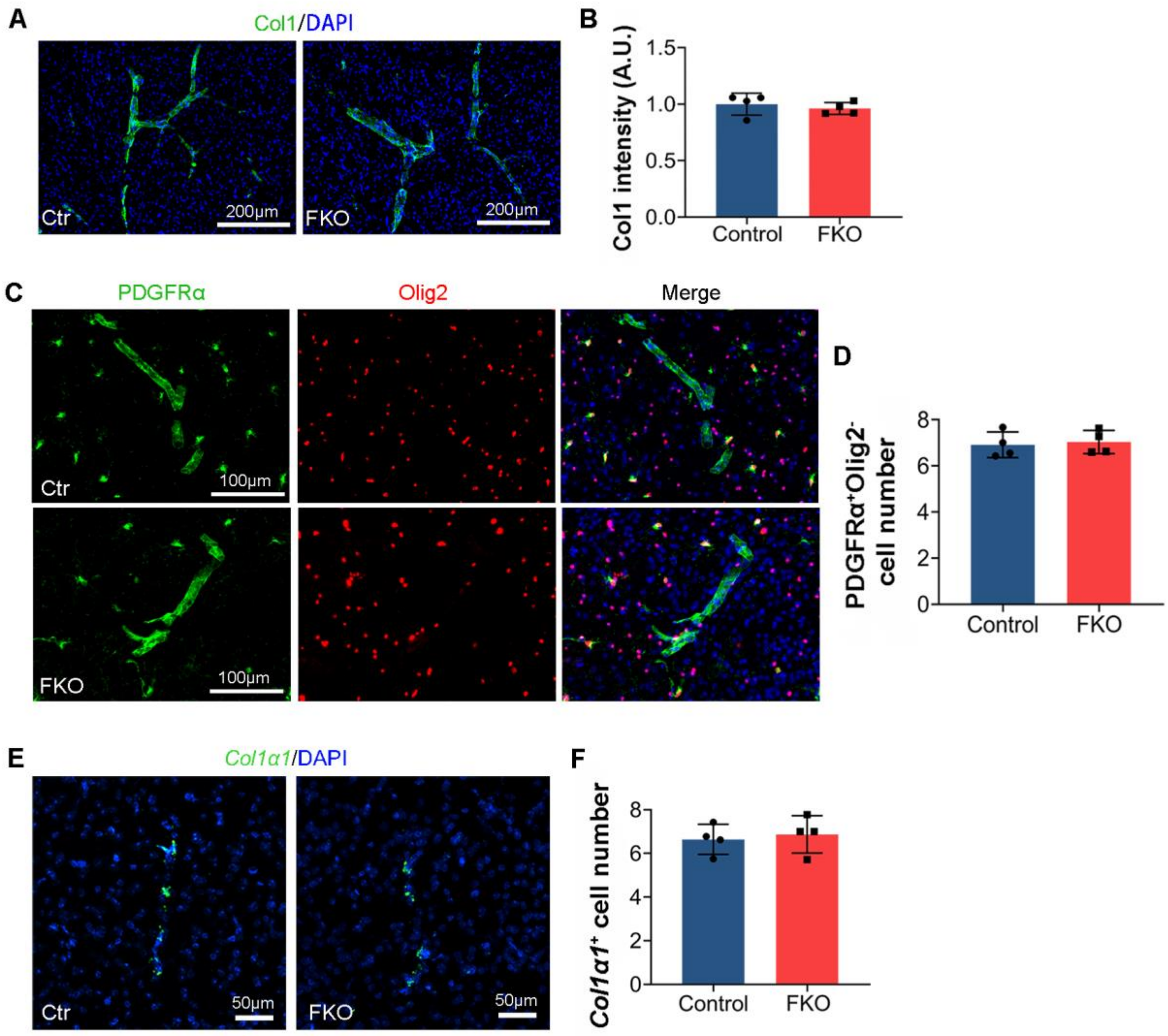
A
Col1/DAPI
Ctr
200μm
FKO
200μm
B
Col1 intensity (A.U.)
1.5
1.0
0.5
0.0
Control
FKO
C
PDGFRα
Olig2
Merge
Ctr
100μm
FKO
100μm
D
PDGFRα⁺Olig2⁻ cell number
8
6
4
2
0
Control
FKO
E
Col1α1/DAPI
Ctr
50μm
FKO
50μm
F
Col1α1⁺ cell number
8
6
4
2
0
Control
FKO

Figure S2. Col1α1^{+} fibroblasts are not ablated in FKO mice under homeostatic conditions with intracerebroventricular injection of DT.

(A) Representative images of Col1 (green) and DAPI (blue) in control and FKO mice under homeostatic conditions with intracerebroventricular injection of DT. Scale bars = 200μm.

(B) Quantification of Col1 intensity in control and FKO brains. n = 4 mice.

(C) Representative images of PDGFRα (green), Olig2 (red), and DAPI (blue) in control and FKO mice under homeostatic conditions with intracerebroventricular injection of DT. Scale bars = 100μm.

(D) Quantification of PDGFRα^{+}Olig2^{-} cells in control and FKO brains. n = 4 mice.

(E) Representative images of *Col1α1* mRNA (green, detected by RNAscope in situ hybridization) and DAPI (blue) in control and FKO mice under homeostatic conditions with intracerebroventricular injection of DT. Scale bars = 50μm.

(F) Quantification of *Col1α1*$^{+}$ cells in control and FKO brains. n = 4 mice.

Data were shown as mean ± SD. Related to Figure 1.

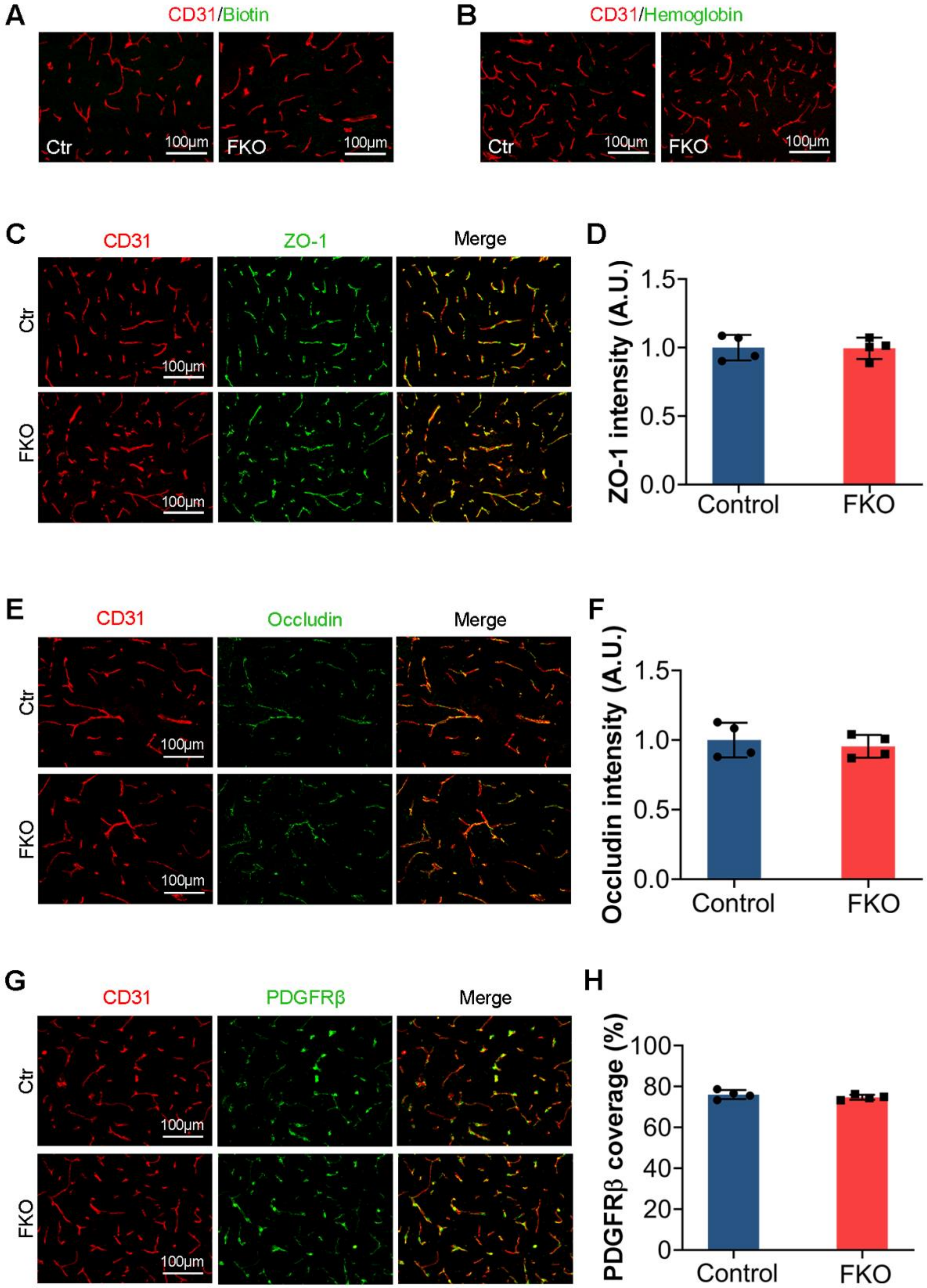
A
CD31/Biotin
Ctr
100μm
FKO
100μm
B
CD31/Hemoglobin
Ctr
100μm
FKO
100μm
C
CD31
ZO-1
Merge
Ctr
FKO
100μm
D
ZO-1 intensity (A.U.)
1.5
1.0
0.5
0.0
Control
FKO
E
CD31
Occludin
Merge
Ctr
FKO
100μm
F
Occludin intensity (A.U.)
1.5
1.0
0.5
0.0
Control
FKO
G
CD31
PDGFRβ
Merge
Ctr
FKO
100μm
H
PDGFRβ coverage (%)
100
80
60
40
20
0
Control
FKO

Figure S3. **BBB integrity is not disrupted in FKO mice under homeostatic conditions.**

(A) Representative images of biotin (green) and CD31 (red) in control and FKO brains under homeostatic conditions. Scale bars = 100μm.

(B) Representative images of hemoglobin (green) and CD31 (red) in control and FKO brains under homeostatic conditions. Scale bars = 100μm.

(C) Representative images of ZO-1 (green) and CD31 (red) in control and FKO brains under homeostatic conditions. Scale bars = 100μm.

(D) Quantification of ZO-1 intensity in control and FKO brains. n = 4 mice.

(E) Representative images of occludin (green) and CD31 (red) in control and FKO brains under homeostatic conditions. Scale bars = 100μm.

(F) Quantification of occludin intensity in control and FKO brains. n = 4 mice.

(G) Representative images of PDGFRβ (green) and CD31 (red) in control and FKO brains under homeostatic conditions. Scale bars = 100μm.

(H) Quantification of PDGFRβ coverage over $CD31^+$ capillaries in control and FKO brains. n = 4 mice.

Data were shown as mean ± SD. Related to Figure 1.

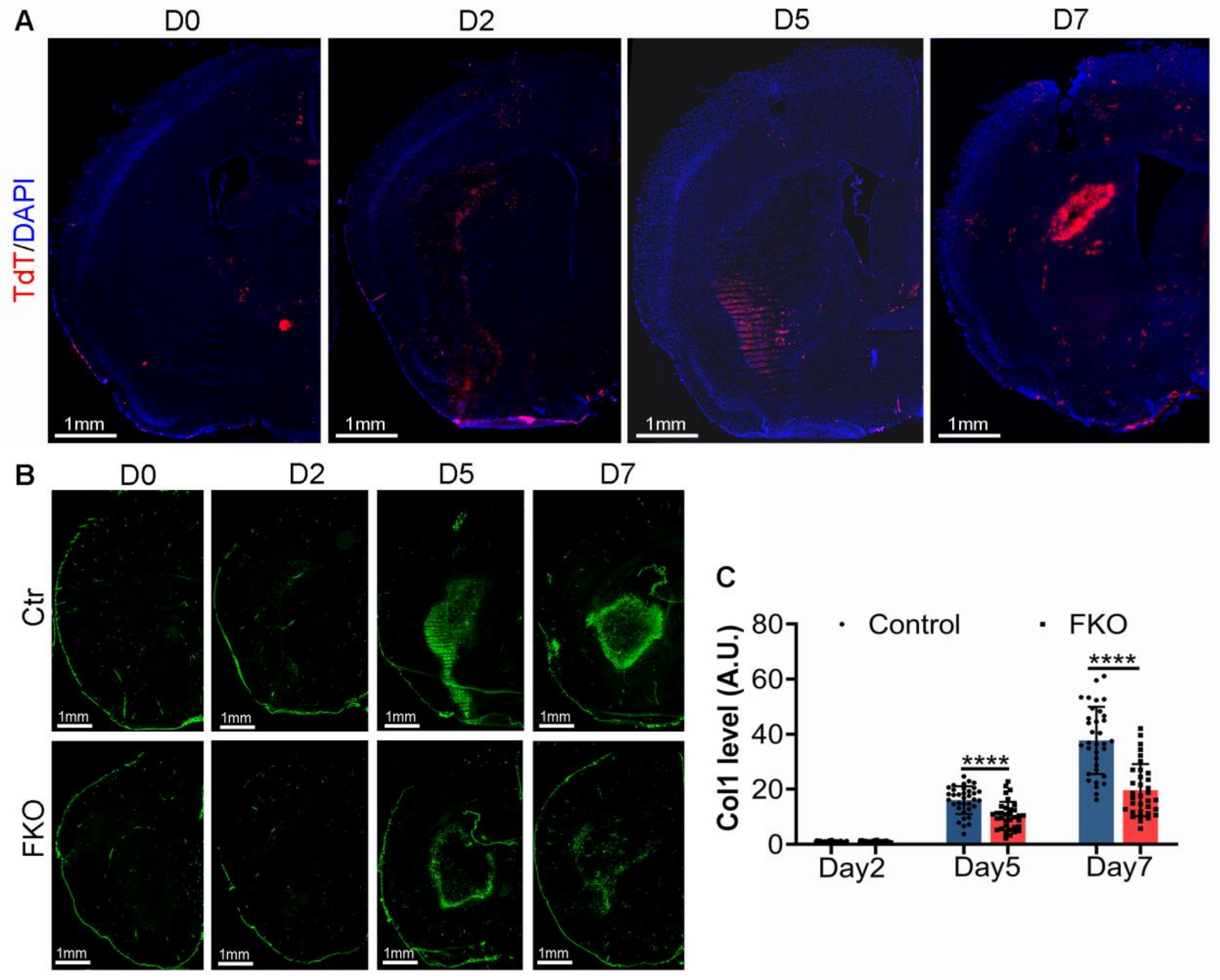
A
D0
D2
D5
D7
TdT/DAPI
1mm
B
D0
D2
D5
D7
Ctr
FKO
1mm
C
Control
FKO
Col1 level (A.U.)
80
60
40
20
0
****
****
Day2
Day5
Day7

Figure S4. Col1α1^{+} fibroblasts accumulate at the injury site after ICH.

(A) Low-magnification images of tdtomato (red) and DAPI (blue) in the brains of Col1α1-tdtomato mice before and at various time points (days 2, 5 and 7) after ICH. Scale bars = 1mm.

(B) Low-magnification images of Col1 (green) in the peri-hematoma region in control and FKO mice before and at various time points (days 2, 5 and 7) after ICH. Scale bars = 1mm.

(C) Quantification of Col1 intensity in the peri-hematoma region in control and FKO mice at days 2, 5, and 7 after ICH. n = 34-36 sections from 4 mice. ****P < 0.0001 by student's t-test.

Data were shown as mean ± SD. ICH, intracerebral hemorrhage. Related to Figures 1 and 2.

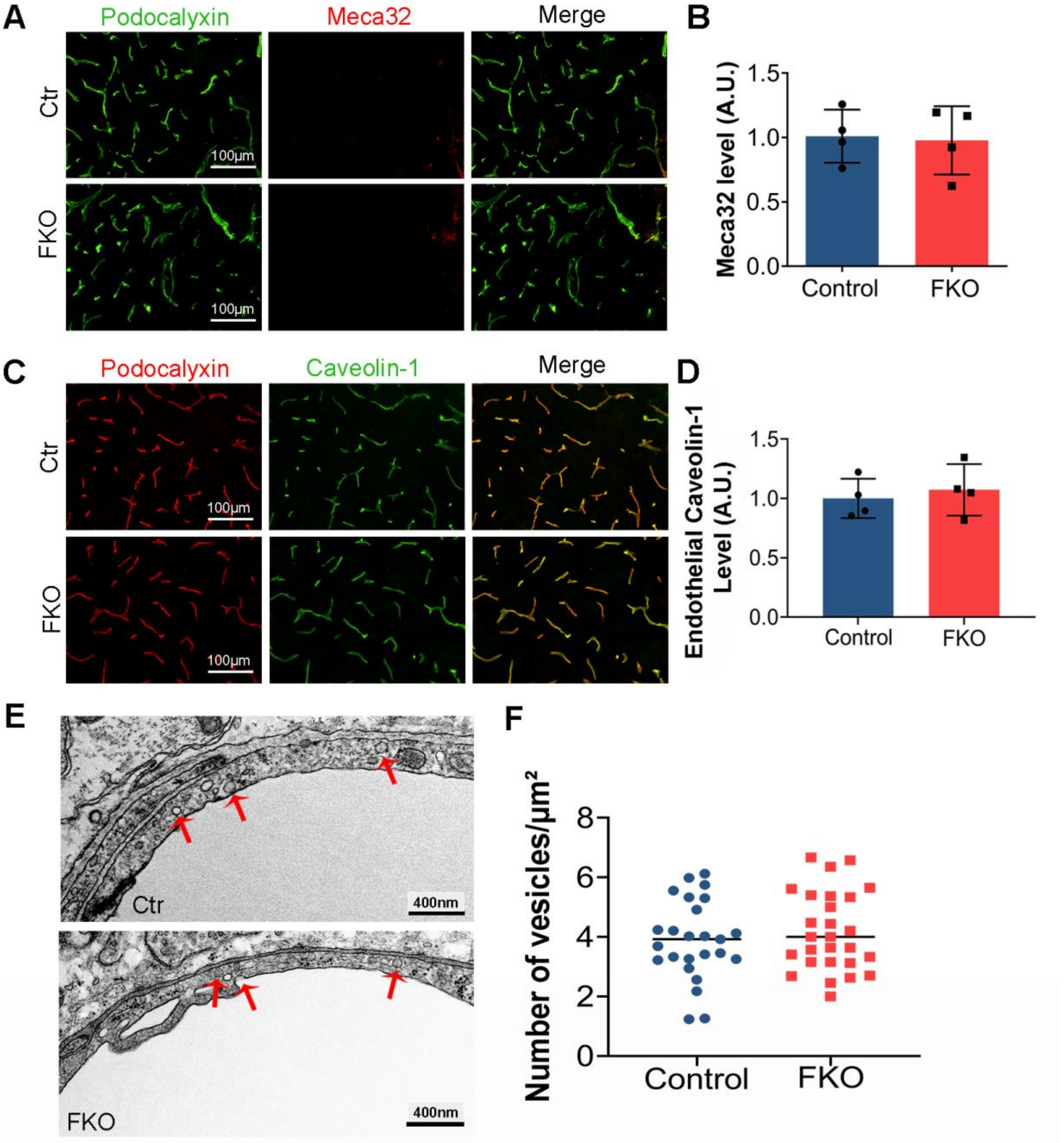

A
Podocalyxin
Meca32
Merge
Ctr
FKO
100µm
B
Meca32 level (A.U.)
1.5
1.0
0.5
0.0
Control
FKO
C
Podocalyxin
Caveolin-1
Merge
D
Endothelial Caveolin-1 Level (A.U.)
E
400nm
F
Number of vesicles/µm²
8
6
4
2
0

Figure S5. Ablation of Col1α1$^+$ fibroblasts does not affect transcytosis at day 7 after ICH.

(A) Representative images of meca32 (red) and podocalyxin (green) in control and FKO mice at day 7 after ICH. Scale bars = 100μm.

(B) Quantification of meca32 intensity in control and FKO brains at day 7 after ICH. n = 4 mice.

(C) Representative images of caveolin-1 (green) and podocalyxin (red) in control and FKO mice at day 7 after ICH. Scale bars = 100μm.

(D) Quantification of endothelial caveolin-1 levels in control and FKO brains at day 7 after ICH. n = 4 mice.

(E) Representative TEM images showing comparable pinocytotic vesicles in capillary endothelial cells from control and FKO brains at day 7 after ICH. Arrows indicate pinocytotic vesicles. Scale bars = 400nm.

(F) Quantification of vesicle number in capillary endothelial cells from control and FKO brains at day 7 after ICH. n = 25-27 capillaries from 3 mice.

Data were shown as mean ± SD. ICH, intracerebral hemorrhage; TEM, transmission electron microscopy. Related to Figure 4.

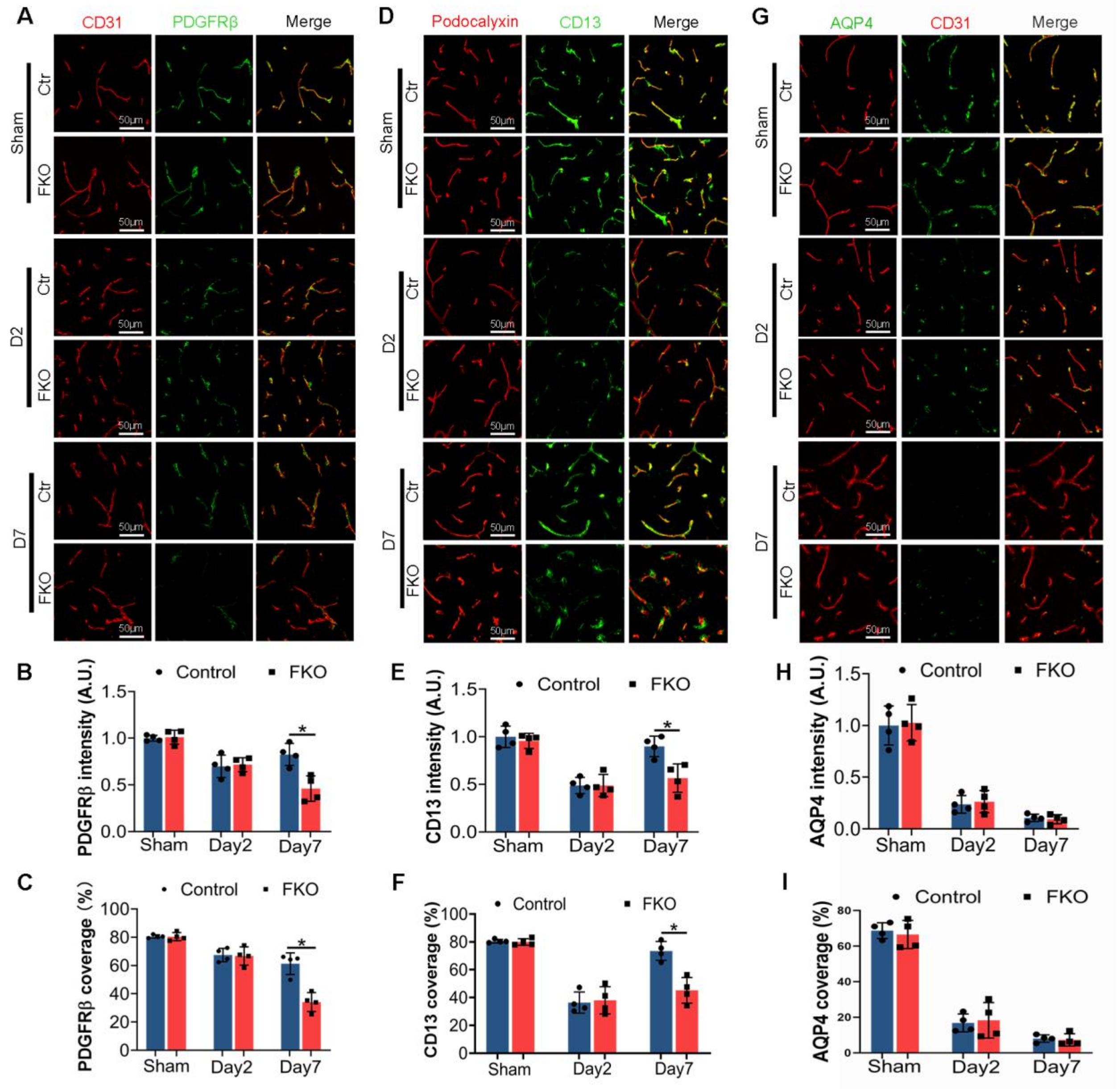

A
CD31
PDGFRβ
Merge
Sham
D2
D7
Ctr
FKO
50μm
D
Podocalyxin
CD13
Merge
G
AQP4
CD31
Merge
B
Control
FKO
PDGFRβ intensity (A.U.)
Sham
Day2
Day7
C
PDGFRβ coverage (%)
E
CD13 intensity (A.U.)
F
CD13 coverage (%)
H
AQP4 intensity (A.U.)
I
AQP4 coverage (%)

Figure S6. The effects of Col1α1^{+} fibroblast ablation on other perivascular cells.

(A) Representative images of PDGFRβ (green) and CD31 (red) in control and FKO brains in sham group and at days 2 and 7 after ICH. Scale bars = 50μm.

(B) Quantification of PDGFRβ intensity in control and FKO brains in sham group and at days 2 and 7 after ICH. n = 4 mice, *p = 0.0286 by Mann-Whitney U test.

(C) Quantification of PDGFRβ coverage over CD31^{+} capillaries in control and FKO brains in sham group and at days 2 and 7 after ICH. n = 4 mice, *p = 0.0286 by Mann-Whitney U test.

(D) Representative images of CD13 (green) and podocalyxin (red) in control and FKO brains in sham group and at days 2 and 7 after ICH. Scale bars = 50μm.

(E) Quantification of CD13 intensity in control and FKO brains in sham group and at days 2 and 7 after ICH. n = 4 mice, *p = 0.0286 by Mann-Whitney U test.

(F) Quantification of CD13 coverage over podocalyxin^{+} capillaries in control and FKO brains in sham group and at days 2 and 7 after ICH. n = 4 mice, *p = 0.0286 by Mann-Whitney U test.

(G) Representative images of AQP4 (green) and CD31 (red) in control and FKO mice in sham group and at days 2 and 7 after ICH. Scale bars = 50μm.

(H) Quantification of AQP4 intensity in control and FKO brains in sham group and at days 2 and 7 after ICH. n = 4 mice.

(I) Quantification of AQP4 coverage over CD31^{+} capillaries in control and FKO brains in sham group and at days 2 and 7 after ICH. n = 4 mice.

Data were shown as mean ± SD. ICH, intracerebral hemorrhage. Related to Figure 4.

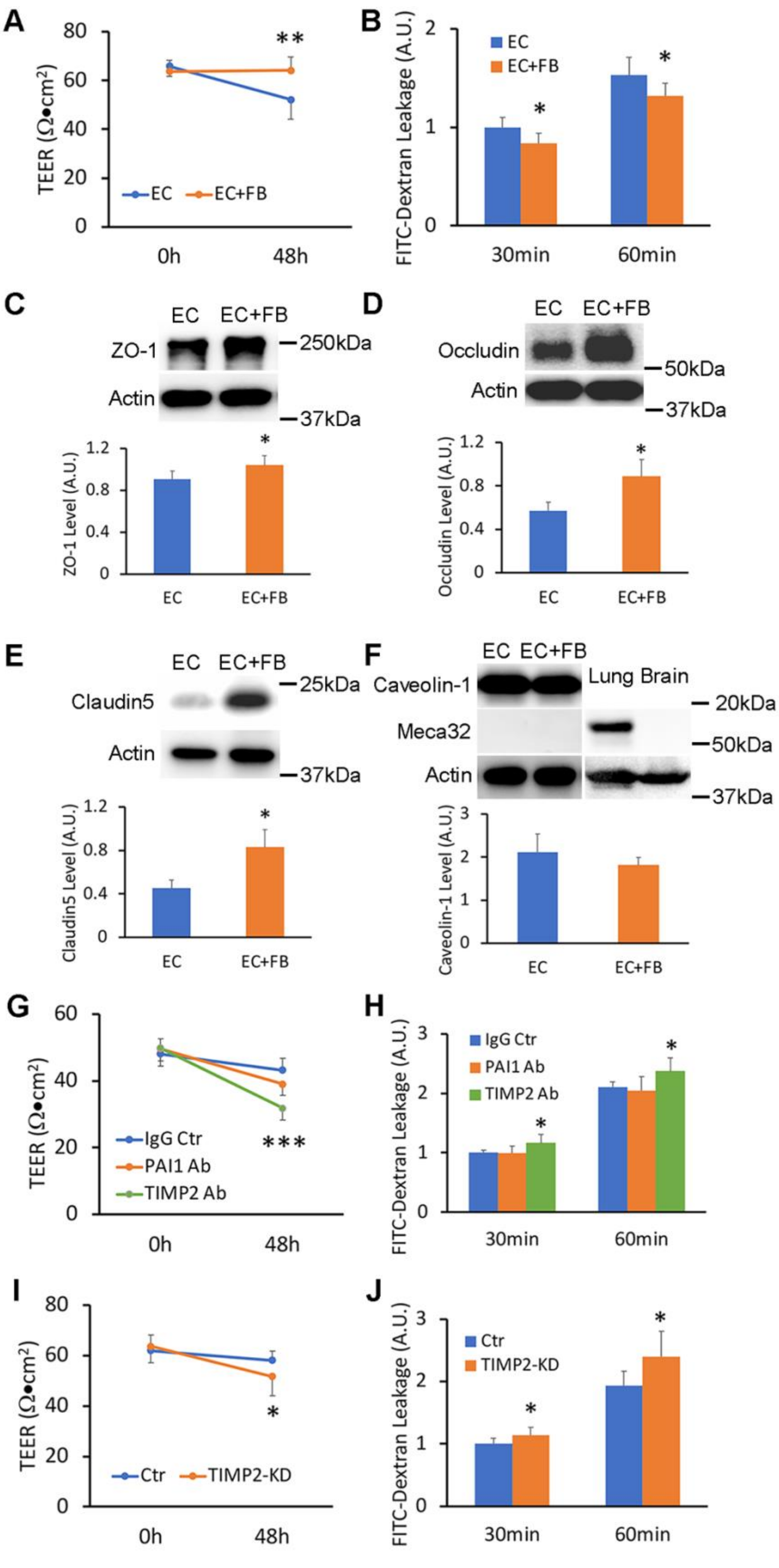

A
TEER (Ω•cm²)
EC
EC+FB
0h
48h
**
B
FITC-Dextran Leakage (A.U.)
EC
EC+FB
30min
60min
C
EC
EC+FB
ZO-1
Actin
250kDa
37kDa
ZO-1 Level (A.U.)
D
EC
EC+FB
Occludin
Actin
50kDa
37kDa
Occludin Level (A.U.)
E
EC
EC+FB
Claudin5
Actin
25kDa
37kDa
Claudin5 Level (A.U.)
F
EC
EC+FB
Lung
Brain
Caveolin-1
Meca32
Actin
20kDa
50kDa
37kDa
Caveolin-1 Level (A.U.)
G
TEER (Ω•cm²)
IgG Ctr
PAI1 Ab
TIMP2 Ab
0h
48h
***
H
FITC-Dextran Leakage (A.U.)
IgG Ctr
PAI1 Ab
TIMP2 Ab
30min
60min
I
TEER (Ω•cm²)
Ctr
TIMP2-KD
0h
48h
J
FITC-Dextran Leakage (A.U.)
Ctr
TIMP2-KD
30min
60min

Figure S7. Fibroblast-derived TIMP2 enhances bEnd3 barrier function in an *in vitro* ICH model.

(A) TEER values of bEnd3 cells with or without fibroblasts. n = 8-9 biological replicates, $^{**}p = 0.0024$ by student's t-test.

(B) Quantification of 4KD-FITC-Dextran leakage in the *in vitro* ICH model. n = 8 biological replicates, $^{*}p = 0.0115$ and $^{*}p = 0.0181$ for 30min and 60min time points by student's t-test, respectively.

(C) Representative western blot images and quantification of ZO-1 from bEnd3 cells cultured with or without fibroblasts for 48 hours after ICH induction. Expression of ZO-1 was normalized to β-actin. n = 5 biological replicates, $^{*}p = 0.0366$ by Mann-Whitney U test.

(D) Representative western blot images and quantification of occludin from bEnd3 cells cultured with or without fibroblasts for 48 hours after ICH induction. Expression of occludin was normalized to β-actin. n = 5 biological replicates, $^{*}p = 0.0121$ by Mann-Whitney U test.

(E) Representative western blot images and quantification of claudin5 from bEnd3 cells cultured with or without fibroblasts for 48 hours after ICH induction. Expression of claudin5 was normalized to β-actin. n = 5 biological replicates, $^{*}p = 0.0121$ by Mann-Whitney U test.

(F) Representative western blot images and quantification of caveolin-1 and meca32 from bEnd3 cells cultured with or without fibroblasts for 48 hours after ICH induction. Lung and brain tissues were used as positive and negative controls for meca32. Expression of caveolin-1 was normalized to β-actin. n = 5 biological replicates.

(G) TEER values of bEnd3 cells co-cultured with fibroblasts in the presence of mouse IgG (control), PAI1 function-blocking antibody, and TIMP2 function-blocking antibody. n = 6 biological replicates, $^{***}p = 0.0003$ by student's t-test.

(H) Quantification of 4KD-FITC-Dextran leakage in the *in vitro* ICH model in the presence of mouse IgG (control), PAI1 function-blocking antibody, and TIMP2 function-blocking antibody. n = 5-6 biological replicates, **p* = 0.0307 and **p* = 0.0367 at 30min and 60min time points by student's t-test, respectively.

(I) TEER values of bEnd3 cells co-cultured with control or TIMP2-KD fibroblasts. n = 10 biological replicates, **p* = 0.0307 by student's t-test.

(J) Quantification of 4KD-FITC-Dextran leakage in the *in vitro* ICH model with control or TIMP2-KD fibroblasts. n = 8 biological replicates, **p* =0.0252 and **p* =0.0159 for 30min and 60min time points by student's t-test, respectively.

Data were shown as mean ± SD. EC, endothelial cells; FB, fibroblasts; KD, knockdown. Related to Figure 6.

Data S1. Unprocessed Western blot images. Related to Figures 6 and S7.

Fig. 6I: TIMP2/Actin

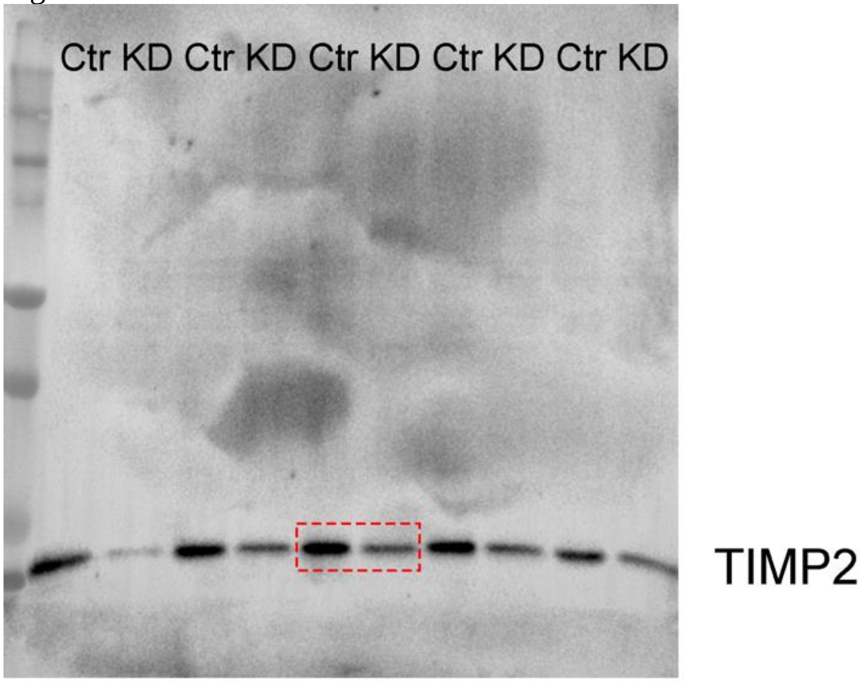

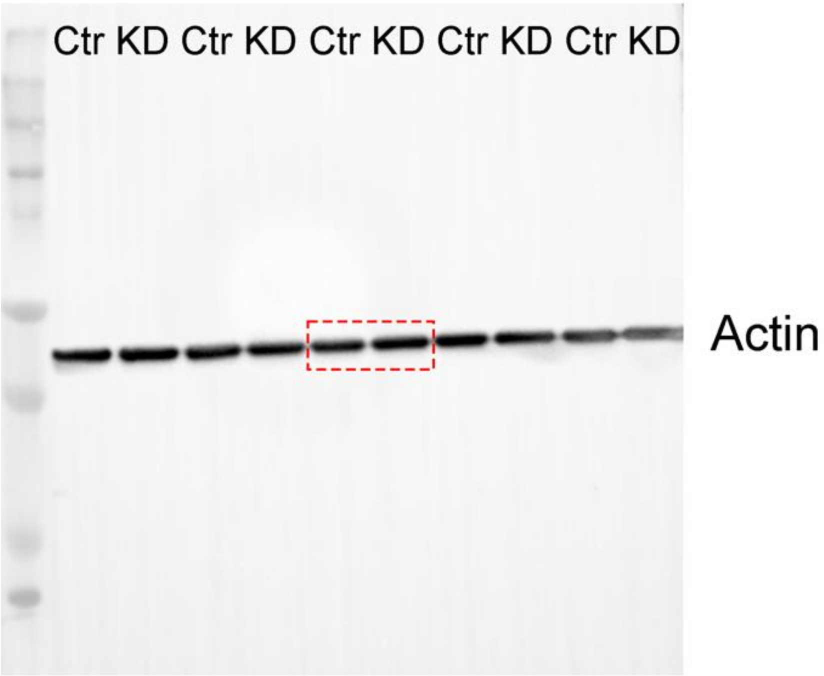

Fig. S7C: ZO-1/Actin

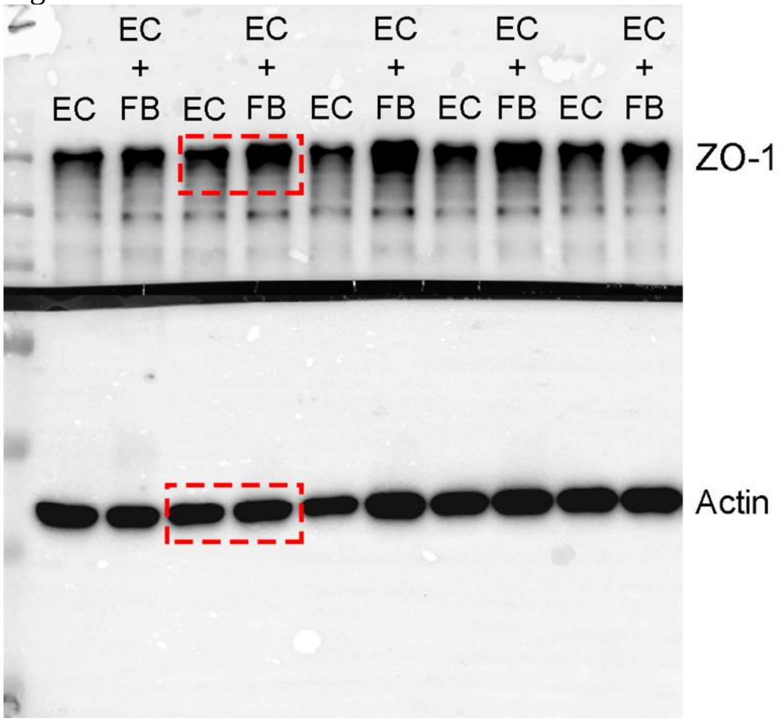

Fig. S7D: Occludin/Actin

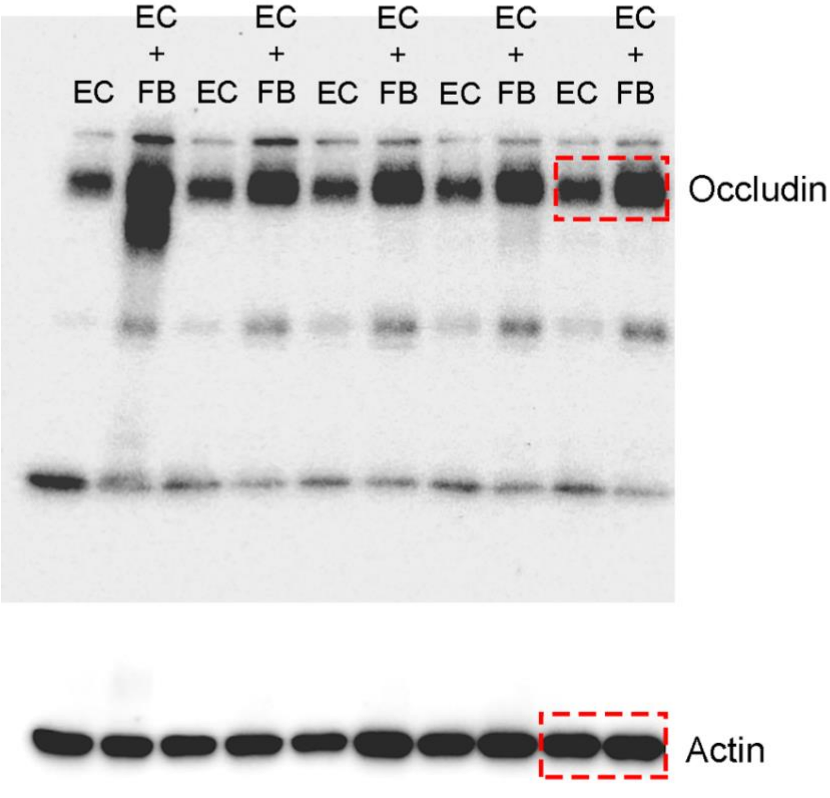

Fig. S7E: Claudin5/Actin

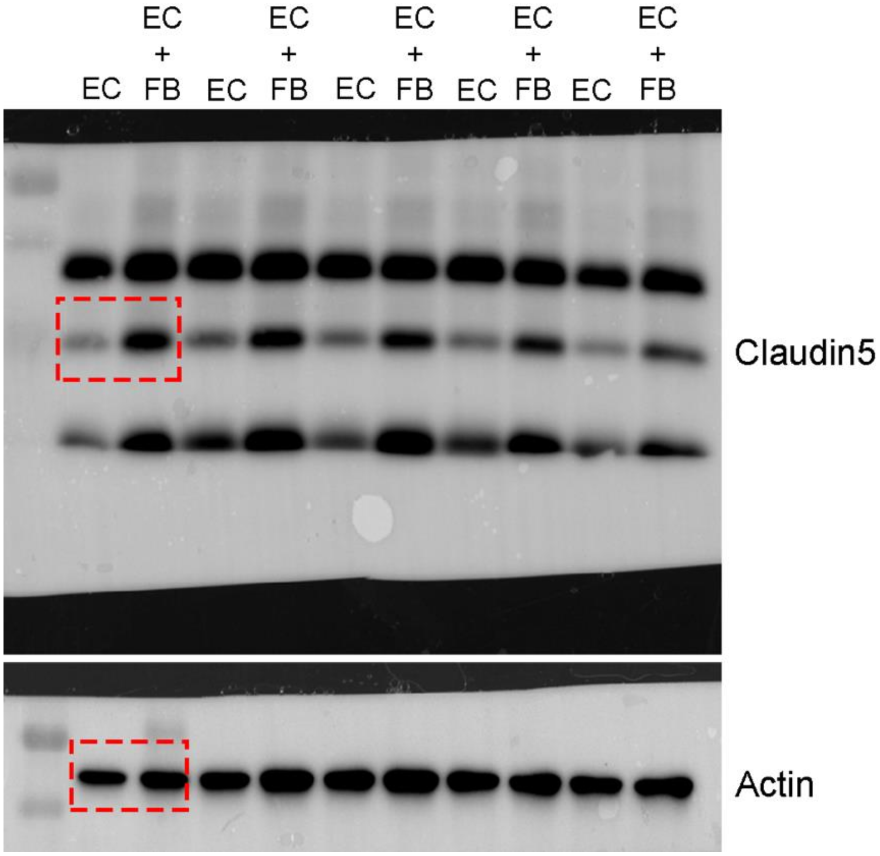

Fig. S7F: Caveolin-1/Meca32/Actin

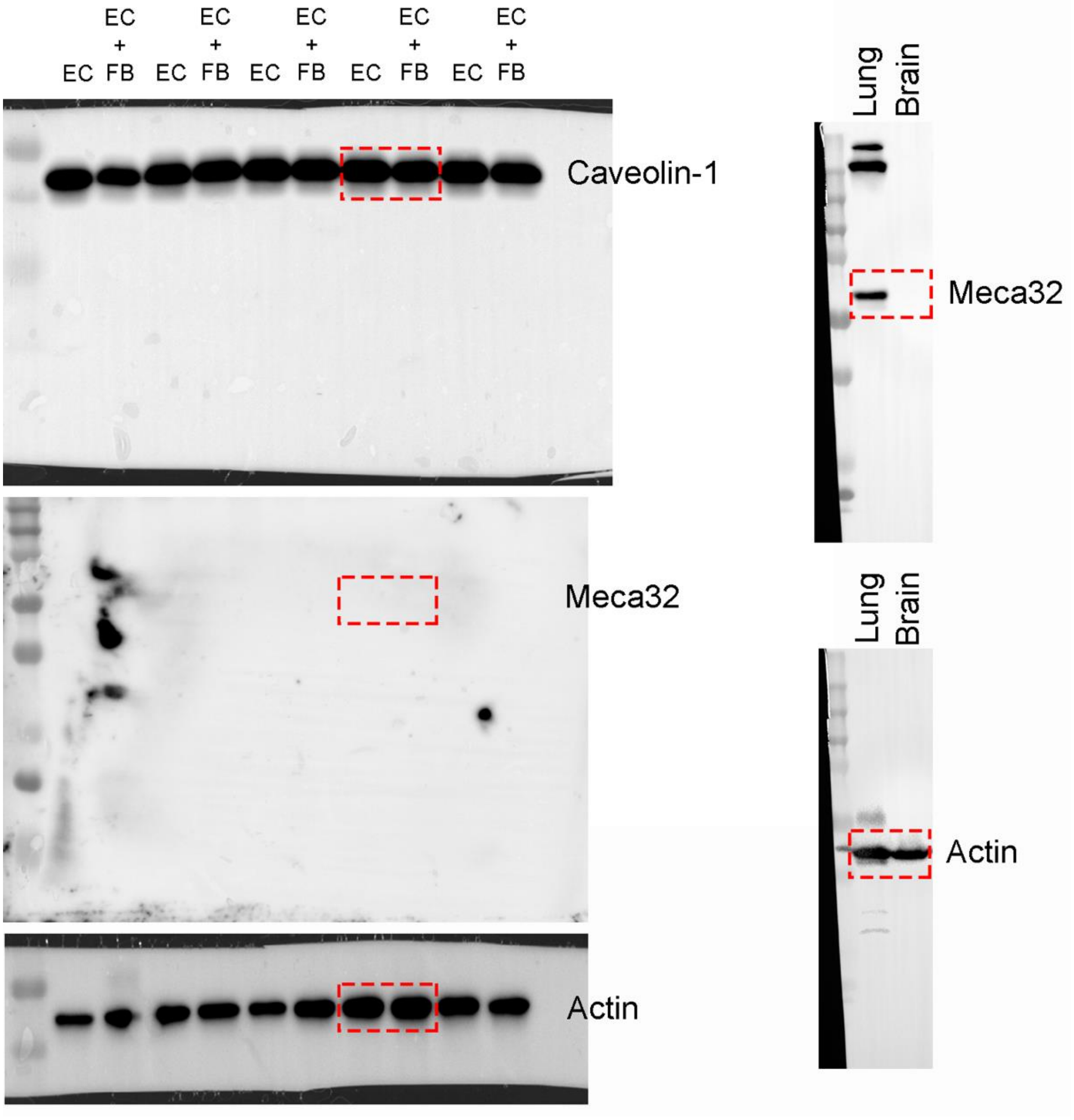

Supplement: 1 [file NIHMS1854754-supplement-1.pdf]
